# Real-World Clinical Oncology Outcomes Associated with the Accelerated Approval Pathway

**DOI:** 10.1158/2767-9764.CRC-25-0225

**Published:** 2026-01-23

**Authors:** Tu My To, Stacey Kowal, James D. Chambers, William B. Wong

**Affiliations:** 1 Genentech, Inc, South San Francisco, California.; 2 Center for the Evaluation of Value and Risk in Health, Institute for Clinical Research and Health Policy Studies, Tufts Medical Center, Boston, Massachusetts.

## Abstract

The accelerated approval (AA) pathway expedites treatment access for patients with unmet medical needs. However, evidence on the program’s impact is lacking. This study assessed real-world clinical outcomes for AA drugs in solid tumor cancers. A nationwide electronic health record (EHR)-derived deidentified database was utilized to explore the impact of the AA pathway in patients with selected solid tumors from 2011 to 2023. Included patients received AA or non-AA products with the same indication. Outcomes were real-world progression-free survival (rwPFS) and overall survival (OS). Population-level health impacts were estimated via market share and real-world progression-free life years (rwPFLYs) and life years (LYs). Twenty-three AA indications were studied. At study completion, 83% of indications had their clinical benefit verified, whereas 17% had been withdrawn from the market. Overall, 78% of indications demonstrated significant improvements in rwPFS or OS, whereas none reported significantly worse rwPFS versus controls. In total, 44% and 48% of indications had >30% rwPFS and OS improvement, respectively. More indications with sample sizes >100 in the AA cohort demonstrated significant rwPFS gains [80% (12/15)] versus cohorts <100 [38% (3/8)]. An estimated 118,107 patients received AA drugs during their AA window, resulting in 56,399 rwPFLYs and 50,848 LYs gained. Most solid tumor AA indications were associated with improved clinical outcomes, providing substantial US population-level health gains. This EHR analysis provides insight into whether the AA pathway appropriately targets promising beneficial therapies. Expanding beyond randomized controlled trial evidence and conventional primary endpoints may be necessary to verify clinical benefit, particularly in rare tumors.

**Significance:**

This study (the first examining the real-world outcomes associated with the AA period) found improved clinical outcomes for most AA drugs studied compared with existing treatments in solid tumor cancers. The improvements observed suggest potential health improvements at the US population level and the possibility of the need to look beyond randomized controlled evidence and traditional primary endpoints to verify the clinical benefit of AA drugs for relatively rare conditions.

## Introduction

Access to medicines in the United States is granted following regulatory approval from the US Food and Drug Administration (FDA), which considers evidence on the safety and efficacy of new drugs based on high-quality clinical data. The FDA’s accelerated approval (AA) pathway was created to expedite access to new treatments with potential benefits over available treatments in serious and life-threatening conditions (1, 2). The AA pathway allows for approval based on a surrogate endpoint, defined as reasonably likely to predict a drug’s clinical benefit (3).

Unlike traditional approval pathways that rely on randomized controlled trial (RCT) data, AA decisions may be based on surrogate endpoints and single-arm trials, with confirmatory trials required to verify treatment benefits (4). The AA pathway brings potentially life-changing treatments to patients with serious conditions quickly; however, risks can occur from treatments with uncertain clinical performance (4).

The AA pathway has faced increased scrutiny in recent years (5). Criticisms include the relevance and validity of surrogate endpoints, delays in completing confirmatory trials, AA products being withdrawn, and the potential economic consequences of withdrawn products (5). Reasons for withdrawals have typically centered around confirmatory studies finding a lack of clinical benefit (6) or evolutions in the treatment landscape (7). Consequently, there have been calls for reforms to the pathway (8), and in March 2023, the FDA issued draft guidance outlining potential reforms (9). As discussions on the future of the AA pathway continue, it is important that these discussions are anchored to robust, relevant evidence on the impact of the program.

Much of the attention on the AA pathway has focused on the consequences of withdrawing products or the outcomes from later pivotal or confirmatory trials, some of which were conducted in different settings or lines of care (10). However, less is known about the ability of the pathway to grant patients earlier access to practice-changing therapies and the associated outcomes. Previous research suggests that earlier access to therapies via the FDA’s expedited review process, including the AA pathway, has led to greater health gains than the conventional approval pathway (3).

Despite increasing evidence on the AA pathway, much of the current information based on modeling techniques or clinical trial summaries may not accurately capture the real-world impact of treatments during their AA window. Given that it is not possible to go back historically and conduct RCTs in this exact environment (the AA window of a medicine), real-world data are needed to draw conclusions on the performance of these medicines during this time period. Thus far, no studies have assessed real-world disease outcomes for patients using AA drugs compared with the standard of care (SoC) in clinical practice. The generation of real-world evidence to complement early trial data and later confirmatory trials is needed to allow for a holistic understanding of the performance of medicines using the AA pathway.

Oncology is an ideal area to explore the real-world outcomes of products approved through the AA pathway (4). The program is most often applied to oncology treatments, and common disease outcomes [such as real-world progression-free survival (rwPFS)] allow for the exploration of health impacts across indications. Additionally, electronic health records (EHRs) provide detailed data, allowing for the consideration of unique patient and disease characteristics to facilitate appropriate comparisons between treatments and SoC. Real-world effectiveness AA drug data are needed to gain a better understanding of whether the AA program works as intended and to know whether early access to these AA drugs leads to clinical benefits in the patients it directly affects. Therefore, this study aimed to evaluate the real-world clinical outcomes associated with the AA pathway in solid tumors during the early-access AA window, at the patient and population level.

## Materials and Methods

### AA drug selection

**Figure 1.**
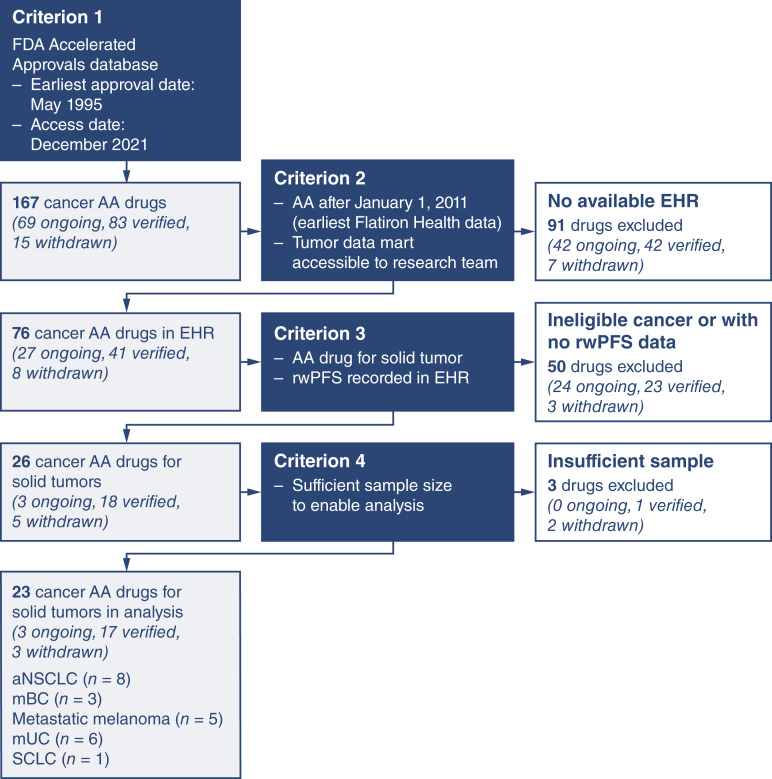
Flow diagram of the study selection process for AA drugs in oncology solid tumors.

A list of all oncology AAs, which have been verified or withdrawn or are ongoing as of December 2021, was compiled from the FDA Accelerated Approvals database (11). From the first available approval in 1995 through to our access date, there were 167 potentially eligible cancer AAs (Fig. 1). This list was refined according to EHR data availability. Specifically, the team isolated AA drugs approved after January 2011 (i.e., the starting date for the EHR data) but before 2020, to allow for sufficient follow-up time to capture progression and mortality outcomes at the time of study initiation (December 2021). In total, 86 AA indications were excluded based on these time requirements, and five indications were excluded due to a lack of access to the tumor data mart for the research team. Next, the sample was restricted to solid tumor cancers in which rwPFS outcome data were available. This key progression outcome measure in the EHR allows for a uniform measure of impact across numerous cancer types. Three drugs were excluded after analysis initiation due to insufficient sample size (i.e., cohort sizes of less than 20 patients). After applying all inclusion criteria, the final sample included 23 drugs: 3 with ongoing AA programs, 17 verified and converted to traditional approval, and 3 withdrawn. Indications were defined as drug-indication pairs, in which each unique indication for a drug was analyzed individually. The final tumor list included advanced non–small cell lung cancer (aNSCLC), metastatic breast cancer (mBC), advanced or metastatic melanoma, advanced or metastatic urothelial cancer (mUC), and small cell lung cancer (SCLC; Table 1; Supplementary Table S1).

**Table 1.** Summary of AA drugs in oncology solid tumors included for real-world analysis.

Included drug	Summary AA indication	AA period (cohort 1)	Comparator period (cohort 2)	Analysis time frame (data cutoff)
Alectinib	ALK+ metastatic NSCLC ≥2L	December 11, 2015–November 6, 2017	November 6, 2012–November 6, 2017	April 30, 2022
Brigatinib	ALK+ metastatic NSCLC ≥2L	April 28, 2017–May 22, 2020	May 22, 2015–May 22, 2020	April 30, 2022
Ceritinib	ALK+ metastatic NSCLC ≥2L	April 29, 2014–May 26, 2017	May 26, 2012–May 26, 2017	April 30, 2022
Crizotinib	ALK+ locally advanced or metastatic NSCLC ≥1L	August 26, 2011–November 20, 2013	January 1, 2011–December 20, 2013	April 30, 2022
Lorlatinib	ALK+ metastatic NSCLC ≥2L	November 2, 2018–March 3, 2021	March 3, 2016–March 3, 2021	April 30, 2022
Pembrolizumab	Metastatic NSCLC ≥1L (PDL1 agnostic)	May 10, 2017–August 20, 2018	May 10, 2011–August 20, 2018	April 30, 2022
Pembrolizumab	PDL1+ metastatic NSCLC ≥2L	October 2, 2015–October 24, 2016	October 9, 2015–October 24, 2016	April 30, 2022
Osimertinib	EGFR+ metastatic NSCLC ≥1L	November 13, 2015–March 30, 2017	January 1, 2011–March 30, 2017	April 30, 2022
Atezolizumab (triple negative)	PDL1+, triple-negative mBC ≥1L	December 20, 2019–May 5, 2022	December 20, 2014–May 5, 2022	June 30, 2023
Palbociclib (ER+, HER2−)	ER+, HER2− advanced breast cancer ≥1L	February 3, 2015–March 31, 2017	January 1, 2011–March 31, 2017	June 30, 2023
Fam-trastuzumab (HER2+)	HER2+ mBC ≥3L	December 20, 2019–May 5, 2022	December 20, 2014–May 5, 2022	June 30, 2023
Dabrafenib	Metastatic melanoma with BRAF *V600E* or *V600K* mutations ≥1L	January 9, 2014–November 20, 2015	January 1, 2011–November 20, 2015	February 28, 2023
Nivolumab plus ipilimumab	Metastatic melanoma ≥1L (BRAF agnostic)	September 30, 2015–March 7, 2019	January 1, 2011–March 7, 2019	February 28, 2023
Nivolumab (BRAF+)	BRAF *V600* mutation-positive metastatic melanoma ≥1L	January 23, 2016–March 7, 2019	January 23, 2011–March 7, 2019	February 28, 2023
Nivolumab	Metastatic melanoma ≥2L	December 22, 2014–March 7, 2019	January 23, 2011–March 7, 2019	February 28, 2023
Pembrolizumab	Metastatic melanoma ≥2L	September 4, 2014–December 18, 2015	January 1, 2011–December 18, 2015	February 28, 2023
Atezolizumab	Locally advanced or mUC ≥1L	April 17, 2017–December 2, 2022	April 17, 2012–December 2, 2022	August 31, 2023
Pembrolizumab	Locally advanced or mUC ≥1L	May 18, 2017–August 31, 2021	March 18, 2012–August 31, 2021	August 31, 2023
Atezolizumab	Locally advanced or mUC ≥2L	May 18, 2016–April 13, 2021	May 18, 2016–April 13, 2021	August 31, 2023
Erdafitinib	Locally advanced or metastatic FGFR+ urothelial cancer ≥2L	April 12, 2019–May 31, 2023	April 12, 2014–May 31, 2023	August 31, 2023
Nivolumab	Locally advanced or mUC ≥2L	February 2, 2017–August 19, 2021	February 2, 2012–August 19, 2021	August 31, 2023
Enfortumab vedotin-ejfv	Locally advanced or mUC ≥2L	December 18, 2019–July 9, 2021	December 18, 2014–July 9, 2021	August 31, 2023
Nivolumab	Metastatic SCLC ≥3L	August 6, 2018–November 29, 2020	August 6, 2018–November 29, 2020	August 31, 2023

Abbreviations: BRAF, v-raf murine sarcoma viral oncogene homolog B1; ER, estrogen receptor; fam-trastuzumab, fam-trastuzumab deruxtecan-nxki; FGFR, fibroblast growth factor receptor; HER2, human epidermal growth factor receptor-2; PDL1, programmed death ligand 1.

### Data source

This study used the nationwide Flatiron Health EHR-derived deidentified database, originating from approximately 280 cancer clinics (∼800 sites of care), most of which were in the community setting. The database comprised longitudinal data from multiple cancer cohorts and consisted of patient-level structured and unstructured data curated via technology-enabled abstraction. It also encompassed a wide range of patient characteristics and documented various aspects of the patient journey, including diagnosis, treatment, and patient outcomes (12). Patients diagnosed with the selected tumor from 2011 (earliest available data) up to June 2023 were included. Patients were aged ≥18 years, had evidence of treatment with systemic therapy in the Flatiron Health network of clinics within 120 days of their advanced/metastatic diagnosis, and had no evidence of multiple tumors. Indication-specific inclusion criteria are available in Supplementary Table S2.

As this research used deidentified secondary data from Flatiron Health, Institutional Review Board review/approval was not required.

### Study design

The study examined patient outcomes during the AA window. For AA drugs (cohort 1), patient-level outcomes were estimated for those who initiated treatment within the AA window, defined as the time between the FDA AA date and the date on which the approval was converted to full approval or was withdrawn. For AAs that were in progress, all available data from the AA to the end of the data availability period were used.

To isolate the incremental impacts of AA drugs, a second cohort examined outcomes for patients with the same tumor who received SoC at the time, defined as any treatment during the time period of interest that was not a product under AA. For many indications, the AA drug quickly became the most commonly used treatment, making the use of a contemporaneous control group subject to channeling bias (13). To ensure sufficient sample size for capturing patient outcomes, a historical counterfactual cohort was created (cohort 2). This included individuals who initiated SoC (i.e., all treatments not approved via AA) during or up to 5 years prior to the AA period for the same indication as the AA product. In some instances, biomarker-driven therapy was the first for that AA indication, and no historical counterfactual cohort was available. This was caused by the absence of biomarker-positive patients and/or a lack of biomarker-positive patients receiving other therapies. In such cases, controls were used from non-biomarker-selected populations. We acknowledge that our approach may not accurately reflect whether the biomarker population is prognostic or predictive for the SoC treatment.

The patient-level outcomes assessed were rwPFS (time from treatment initiation to real-world progression or death) and overall survival (OS; time from treatment initiation to death). Real-world progression in the data was anchored on abstracted clinician documentation, which stemmed from the results of diagnostic procedures and tests, such as radiology and pathology reports. The date of progression was defined as the date with evidence of progression as noted by the clinician (e.g., through a pathology report) or the date the clinician noted progression if no source evidence was available. The date of death was derived from three different data sources: the EHR, Social Security Death Index, or obituary data. To mitigate the misclassification of patients’ event status, patients without documented progression and/or a date of death were censored at the date of the last clinic visit for rwPFS and at the last confirmed activity for OS. Individuals with rwPFS or OS time less than 14 days were removed from the analyses because their time at risk and any event captured would be unrelated to their treatment status. The methodology of abstraction and validation of real-world progression has been described previously (14–16).

We also sought to understand the population-level impact of AA drugs during the AA window, which considered the real-world uptake of these drugs. Specifically, rwPFS and OS outcomes reaching statistical significance were extrapolated to the US population level using national estimates of the number of patients per tumor and therapy line from CancerMPact (Supplementary Table S3; ref. 17). This information was combined with market share estimates from the Flatiron Health database to estimate the impact of the AA pathway on total real-world progression-free life years (rwPFLYs) and life years (LYs). A sensitivity analysis included nonsignificant differences in rwPFS or OS in estimating total health impacts at the US level.

### Statistical analyses

Descriptive statistics were used to characterize baseline demographics. Restricted mean rwPFS and OS were estimated using stabilized inverse probability of treatment weighted (IPTW) Kaplan–Meier curves. The follow-up duration for cohort 2 could exceed the duration of follow-up for cohort 1 and could bias the estimation of the restricted means. Owing to this, survival times were truncated for the controls that exceeded the total time of data availability for cohort 1 (cohort selection to last data cutoff date). Marginal hazard ratios (HRs) and 95% confidence intervals (CIs) were estimated using stabilized IPTW Cox proportional hazard models. With IPTW, individuals in the analysis were weighted by the inverse of the probability of receiving the treatment of interest (i.e., the inverse of their propensity score for treatment), conditional on various baseline characteristics that were determined *a priori* to be potential confounders. This reduced bias due to confounding by balancing baseline characteristics between the AA and control cohorts. To mitigate the issue of extreme weights, stabilized IPTW was used. IPTW included age, sex, race/ethnicity, and region for all models. Additionally, indication/tumor-specific characteristics were included in constructing the weights when available, including histology, oncologist-defined rule-based therapy line, recurrence, number of prior specific therapies [i.e., anaplastic lymphoma kinase (ALK) inhibitors], primary site, Eastern Cooperative Group Performance Score, and stage at initial diagnosis. Covariate missingness was included as a separate category when calculating the IPTW. We visually assessed the proportional hazards assumption using log–log plots. Time-partitioned weighted Cox models were estimated when the proportional hazards assumption was violated. Analyses used R version 4.1.3.

## Results

### Sample selection

This study examined 23 AA solid tumor indications: aNSCLC (8), mBC (3), advanced/metastatic melanoma (5), mUC (6), and SCLC (Table 1; see Supplementary Tables S4–S8 for baseline characteristics; ref. 1). At study initiation, 17 indications had been verified, 3 were ongoing, and 3 were withdrawn (Fig. 1). Thirteen indications (54%) required biomarker testing to confirm the presence or absence of a biomarker.

### rwPFS and OS

**Figure 2.**
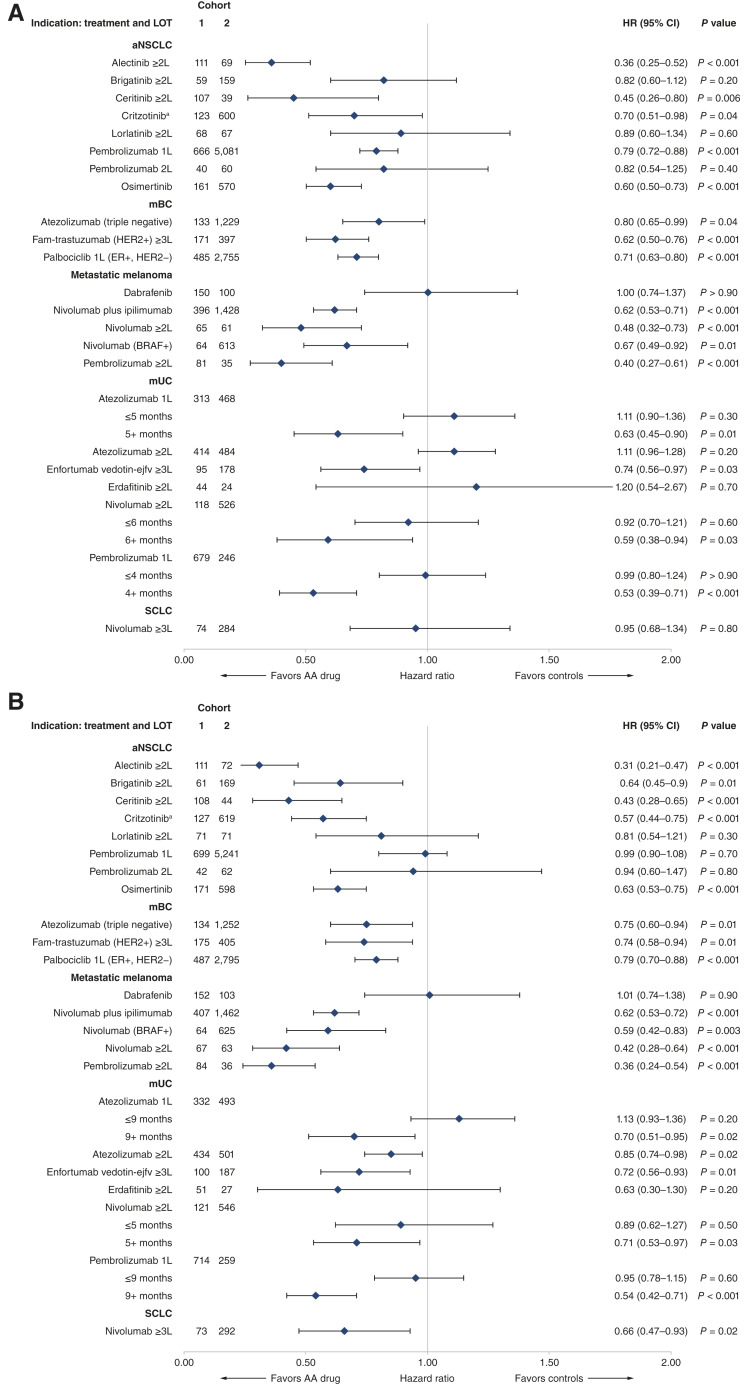
Risk of progression or death (**A**) and risk of death (**B**) for AA drugs in oncology solid tumors vs. controls. ^a^The historical control was not restricted to the ALK+ population. Crizotinib was the first ALK inhibitor and, therefore, there was no information on ALK status prior to crizotinib being available. BRAF, v-raf murine sarcoma viral oncogene homolog B1; CI, confidence interval; ER, estrogen receptor; fam-trastuzumab, fam-trastuzumab deruxtecan-nxki; HER2, human epidermal growth factor receptor-2; LOT, line of therapy.

Of the 23 indications examined, 15 (65%) demonstrated evidence of statistically significant improvement in rwPFS (Fig. 2A, see Supplementary Table S9 for unweighted estimates). For indications requiring biomarker testing, eight (62%) indications showed significant rwPFS gains (Fig. 2A). Four indications (17%; two biomarkers and two cancer immunotherapies) demonstrated >50% reductions in the risk of real-world progression or death, six (26%) demonstrated 30% to 50% reductions, and five (22%) demonstrated 20% to 30% reductions. Notably, no indications demonstrated significantly worse rwPFS than controls. More indications with sample sizes >100 in cohort 1 demonstrated significant gains [80% (12/15)] compared with those with sample sizes <100 in cohort 1 [38% (3/8)].

Findings showing significant improvement in OS were similar [65% (15/23)], including nine among indications requiring biomarker testing (69%; Fig. 2B). Four indications (17%; two biomarker indications and two cancer immunotherapies) demonstrated >50% reductions in the risk of death, seven (30%) demonstrated 30% to 50% reductions, and four (17%) demonstrated 20% to 30% reductions. Similar to findings for rwPFS, no AA indications demonstrated significantly worse OS compared with controls. Once again, more indications with sample sizes >100 in cohort 1 demonstrated significant gains [67% (10/15)] compared with those with sample sizes <100 in cohort 2 [63% (5/8)].

### Comparison of real-world evidence to FDA decisions

At the time of study completion, 19 (83%) of the studied indications had been verified, and four (17%) had been withdrawn (Table 2). A similar percentage of indications were found to have significant improvements in either rwPFS or OS [78% (18/23)]. Among the four withdrawn indications, one had neither significant rwPFS nor OS, two had significant OS or PFS, and one had both significant rwPFS and OS (Table 2). Among the 19 verified indications, 15 (79%) had a significant improvement in rwPFS or OS. Among verified indications without significant improvements in either rwPFS or OS, the sample sizes in the analysis cohorts were smaller than in the clinical trials, which could explain the lack of statistically significant outcomes.

**Table 2.** Comparison of real-world evidence findings and FDA decisions for oncology solid tumors.^a^

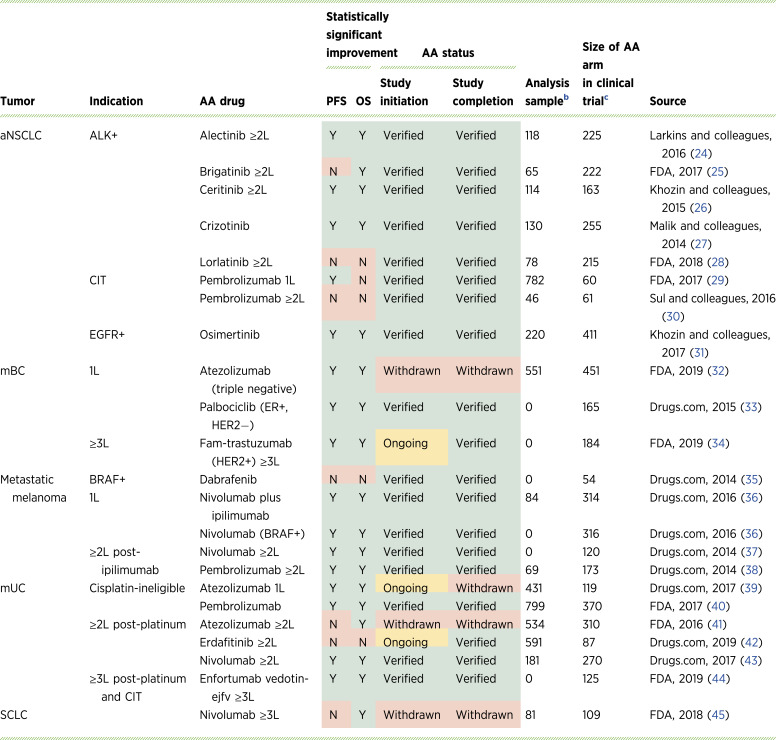

Abbreviations: BRAF, v-raf murine sarcoma viral oncogene homolog B1; CIT, cancer immunotherapy; ER, estrogen receptor; fam-trastuzumab, fam-trastuzumab deruxtecan-nxki; HER2, human epidermal growth factor receptor-2; mUC, advanced or metastatic urethral cancer.

a Three products had ongoing AA when the study was initiated but now have an updated status: atezolizumab for mUC was withdrawn on August 11, 2024; erdafitinib for mUC was verified on January 19, 2024; and fam-trastuzumab was verified on April 5, 2022.

b Number of patients on the AA product in the real-world data analysis from which outcomes were assessed.

c Number of patients on the AA drug in the clinical trial used to grant the AA.

### Population-level estimates of rwPFS and OS gains associated with AA indications

The largest gains in rwPFS and OS for AA products versus SoC were for pembrolizumab second-line (2L) or greater for melanoma (rwPFS, 24.1 months; OS, 30.9 months), nivolumab ≥2L for melanoma (rwPFS, 18.7 months; OS, 25.9 months), and alectinib ≥2L for NSCLC (rwPFS, 15.5 months; OS, 26.1 months; Table 3; Supplementary Table S10). At the population level, an estimated 118,107 patients were treated with these AA drugs during the AA window, resulting in 56,399 rwPFLYs gained and an estimated 50,848 LYs gained. The results were similar in a sensitivity analysis including nonsignificant estimates of rwPFS and OS in the population estimates, with an estimated 55,549 rwPFLYs and 50,367 LYs gained.

**Table 3.** Patient and population outcomes during AA window for drugs in oncology solid tumors.

Tumor	AA drug	Patient-level outcomes^a^		Population treated with AA drug	Population health impacts		Population health impacts (sensitivity analysis)	
		PFS difference	OS difference		PFS years gained	OS years gained	PFS years gained	OS years gained
aNSCLC	Alectinib ≥2L	15.5	26.1	2,055	2,661	4,470	2,661	4,470
	Brigatinib ≥2L	−0.3	8.3	1,269	—	877	(32)	877
	Ceritinib ≥2L	4.6	18.7	2,064	793	3,216	793	3,216
	Crizotinib	7.3	18	3,194	1,938	4,791	1,938	4,791
	Lorlatinib ≥2L	0.8	2.5	1,806	—	—	120	376
	Pembrolizumab 1L	4.2	0.4	27,198	9,519	—	9,519	907
	Pembrolizumab ≥2L	3.3	0.9	7,037	—	—	1,935	528
	Osimertinib	5.7	9.9	3,030	1,447	2,500	1,447	2,500
mBC	Atezolizumab (triple negative)	3.4	4.8	3,772	1,062	1,509	1,062	1,509
	Fam-trastuzumab (HER2+) ≥3L	5	3.6	1,815	756	544	756	544
	Palbociclib (ER+, HER2−)	9.8	7.3	15,053	12,293	9,157	12,293	9,157
Metastatic melanoma	Dabrafenib	−6.7	−6.1	2,845	—	—	(1,579)	(1,446)
	Nivolumab plus ipilimumab	15.1	11.7	8,454	10,638	8,242	10,638	8,242
	Nivolumab (BRAF+)	12.4	14.7	2,013	2,080	2,466	2,080	2,466
	Nivolumab ≥2L	18.7	25.9	1,386	2,160	2,992	2,160	2,992
	Pembrolizumab ≥2L	24.1	30.9	1,596	3,203	4,115	3,203	4,115
mUC	Atezolizumab 1L	0.6	−1.8	7,733	387	—	387	(1,160)
	Atezolizumab ≥2L	−2.4	0.3	7,193	—	—	(1,439)	180
	Enfortumab vedotin-ejfv ≥3L	−0.1	2.9	955	—	231	(6)	231
	Erdafitinib ≥2L	−6.2	−5.2	708	—	—	(366)	(307)
	Nivolumab ≥2L	5.9	2.3	2,308	1,135	—	1,135	442
	Pembrolizumab	5.9	4.6	12,870	6,328	4,933	6,328	4,933
SCLC	Nivolumab ≥3L	3.5	5.5	1,754	—	804	515	804
Total population impact		​	​	118,107	56,399	50,848	55,549	50,367

Abbreviations: BRAF, v-raf murine sarcoma viral oncogene homolog B1; ER, estrogen receptor; fam-trastuzumab, fam-trastuzumab deruxtecan-nxki; HER2, human epidermal growth factor receptor-2; mUC, advanced or metastatic urethral cancer.

a Reported as differences in restricted mean months.

## Discussion

In this study utilizing EHR data, we found that the majority of AA indications in our subset of solid tumors were associated with improvements in real-world clinical outcomes. For the 23 solid tumor indications included, an estimated 118,107 patients accessed AA products during their early approval window, leading to more than 56,000 rwPFLYs and almost 51,000 LYs gained in the United States. Furthermore, most verified indications were associated with improvements in rwPFS and OS; for those that did not, sample sizes were smaller than in the clinical trial. To our knowledge, this is the first study examining the real-world outcomes associated with the AA period.

There have been no other published real-world assessments of the clinical effectiveness of drugs approved under the AA pathway based on patient-level outcomes. However, several studies have examined the clinical outcomes of the AA pathway using alternative methods. One analysis of 46 oncology AA indications examined trends in OS based on confirmatory clinical trials, finding that less than half demonstrated clinical benefit, in which the authors categorized the absence of a confirmatory trial as no clinical benefit (18). For the subset of indications with reported trial outcomes, 69% had a demonstrated benefit in OS or quality of life. This estimate was lower than our finding of around 80% of AA indications showing an OS benefit. However, direct comparison between studies is challenging owing to differences in included oncology indications and the use of confirmatory trials to ascertain benefit, because not all trials were in the AA indication and comparators were not representative of the exhaustive SoC treatments used in the real-world setting. Notably, only 38% of the confirmatory clinical trials in the sample from the other published study were in the same indication as the AA (18). This highlights that the pace of change in clinical practice is so rapid in some cancers that relevant comparators in a real-world evidence context may not be well matched to comparisons in confirmatory studies.

Another study modeled incremental outcomes from clinical trials to estimate US population effects of AA drugs, concluding that >900,000 patients used these products from 2006 to 2022 during their AA window, resulting in 263,000 additional LYs (19). Although our findings also indicate positive outcomes at the population level, we estimated fewer patients receiving AA drugs and fewer OS gains. This difference was likely due to the smaller number of indications in our analysis (23 vs. 69), the smaller patient sample size, a focus on several smaller tumors (i.e., ALK+), and using Flatiron Health to estimate tumor type-specific market shares, lines of therapy, and biomarker status.

Although no AA drugs were found to have significantly worse outcomes than SoC in our subset of solid tumors, some drugs were indeed withdrawn. However, our findings are consistent with confirmatory trials for these withdrawn indications, because none showed that the AA drug had worse outcomes compared with the control. There are various reasons why the real-world data results described here may not align with an outcome of withdrawal of the AA indication. Sometimes, indications or combination therapies in the confirmatory trial differed from the initial trial for which AA was granted, which could explain improvements in outcomes found in our study for withdrawn drugs. For example, this was the case for nivolumab in SCLC, in which the original AA indication was for use in patients in third line or later (≥3L), whereas the confirmatory trials evaluated its use in first-line (1L) and 2L settings. Differences between real-world and trial populations, as well as control arm treatments, are additional explanations. Lastly, nonefficacy-related reasons may also play a role in withdrawing indications, such as the evolution of the treatment landscape, which has been cited for not maintaining the AA indication, as exemplified by the case of atezolizumab for triple-negative breast cancer (7).

Within our sample, we found that analyses with smaller sample sizes (including those smaller than the trial) were less likely to have a significant rwPFS or OS gain compared with analyses with larger sample sizes. Some nonsignificant results may reflect limited sample sizes in our EHR-based analyses rather than an absence of clinical benefit. This may explain why some findings were nonsignificant despite the clinical benefit being verified. Although real-world data hold promise in determining clinical benefit, rare tumors and orphan conditions pose a challenge due to sample sizes. Expedited review pathways, including the AA pathway, have been particularly useful in bringing treatments for rare diseases to market (20). Relying on traditional endpoints to verify clinical benefit (such as OS) for these treatments may be impractical, even with the use of real-world data. Our study supports the consideration of nontraditional endpoints and data sources beyond RCTs as being clinically relevant and meaningful in assessing clinical benefit.

### Limitations

Although RCTs are the gold standard in assessing efficacy through the elimination of confounding, real-world data analyses can be critical in answering research questions such as the present one, in which RCTs are impractical or infeasible to conduct. That being said, careful consideration must be taken to reduce confounding to measure real-world effectiveness. In this analysis, we used IPTW Kaplan–Meier curves to adjust for bias due to confounding factors between historical controls and the AA cohort. However, there is potential for residual confounding effects that may bias findings (21). As the potential for residual bias remains, it is important that insights from this real-world analysis be viewed alongside early trial and later confirmatory RCTs when drawing conclusions on the efficacy and effectiveness of these AA treatments. Although we attempted to take a broad examination of the AA pathway across multiple indications, we were unable to examine all oncology indications. This was in part due to data availability, although many of the excluded indications were rare tumors with limited sample sizes. Next, the potential for postprogression treatments may have advanced over time, which may bias the OS in favor of the AA cohort. Furthermore, although we attempted to control for many factors, some limitations remain about our control group. For example, in the case of the AA drug being the first in a biomarker-selected population, we used a non-biomarker–selected population as the control, given that there were limited biomarker-positive patients who did not receive the AA drug. Nonetheless, the use of a non-biomarker-selected population as the control aligns with the counterfactual scenario, and if the biomarker is not prognostic, this would have a limited impact on our analysis. Additionally, the generalizability of the study findings to oncology indications beyond solid tumor cancers is uncertain. Further research using other databases across more indications is warranted. Furthermore, our study timeline also encompassed the peak COVID-19 period (2020–2021), which could present challenges in capturing progression outcomes requiring interaction with the healthcare setting. However, many of the selection/follow-up periods were outside this peak, and mortality data were derived from external sources (e.g., vital statistics, obituary records) in which reporting may have been affected less. Lastly, this study examined outcomes during the AA window. However, clinical impacts may change as clinical practice evolves after the AA period ends. Confirmatory evidence and real-world data after traditional approval are still needed to understand how the effectiveness of AA drugs changes over time to ascertain whether they continue to bring meaningful clinical benefit (22, 23).

### Conclusion

The AA pathway provided clinical benefits in real-world settings most of the time for the included subset of solid tumor cancers, with no indications performing significantly worse than existing treatments. For the 23 solid tumor indications studied, our findings suggest that earlier access to drugs through the AA pathway may yield meaningful population health gains in the United States.

## Supplementary Material

Supplementary Table S1 Table S1. Additional details of AA indications and dates for drugs in oncology solid tumors included for analysis

Supplementary Table S2 Table S2. Indication-specific inclusion criteria for analysis of outcomes of AA drugs in oncology solid tumors

Supplementary Table S3 Table S3. Additional detail on population impact extrapolation

Supplementary Table S4 Table S4. Baseline patient characteristics among those with aNSCLC

Supplementary Table S5 Table S5. Baseline patient characteristics among those with mBC

Supplementary Table S6 Table S6. Baseline Patient Characteristics Among Those With Melanoma

Supplementary Table S7 Table S7. Baseline patient characteristics among those with mUC

Supplementary Table S8 Table S8. Baseline patient characteristics among those with SCLC

Supplementary Table S9 Table S9. Unweighted risk of progression or death and risk of death for AA drugs vs SoC

Supplementary Table S10 Table S10. Detailed analysis of outcomes for AA drugs in oncology solid tumors

## Data Availability

The data that support the findings of this study were originated by and are the property of Flatiron Health, Inc. Additional data, including Kaplan–Meier curves, are available upon request. Requests for data sharing by license or by permission for the specific purpose of replicating results in this manuscript can be submitted to PublicationsDataAccess@flatiron.com.
