# Supplementary material for: Real-World Clinical Oncology Outcomes Associated with the Accelerated Approval Pathway
Source: Cancer Res Commun. 2026 Jan 23;6(1):191–200. doi: 10.1158/2767-9764.CRC-25-0225 (PMC12828896; doi:10.1158/2767-9764.CRC-25-0225)
Supplement: Supplementary Table S1 — Table S1. Additional details of AA indications and dates for drugs in oncology solid tumors included for analysis [file crc-25-0225_supplementary_table_s1_suppst1.docx]

**Supplementary Table S1.** Additional details of AA indications and dates for drugs in oncology solid tumors included for analysis

| **Drug** | **AA indication** | **AA date** |
| --- | --- | --- |
| Alectinib ≥2L | ALK+ metastatic NSCLC that progressed on or is intolerant to crizotinib | 12/11/2015 |
| Brigatinib ≥2L | ALK+ metastatic NSCLC that has progressed or is intolerant to crizotinib | 4/28/2017 |
| Ceritinib ≥2L | ALK+ metastatic NSCLC that progressed on or is intolerant to crizotinib | 4/29/2014 |
| Crizotinib | Locally advanced or metastatic NSCLC that is ALK+ as detected by an FDA-approved test | 8/26/2011 |
| Lorlatinib ≥2L | ALK+ metastatic NSCLC that has progressed on:   - crizotinib and at least one other ALK inhibitor for metastatic disease - alectinib as the first ALK inhibitor therapy for metastatic disease - ceritinib as the first ALK inhibitor therapy for metastatic disease | 11/2/2018 |
| Pembrolizumab 1L | Metastatic non-squamous NSCLC, in combination with pemetrexed and carboplatin | 5/10/2017 |
| Pembrolizumab ≥2L | Metastatic PD-L1+ NSCLC, as determined by an FDA-approved test, with progression on or after platinum-containing chemotherapy | 10/2/2015 |
| Osimertinib | Metastatic EGFR *T790M* mutation-positive NSCLC, as detected by an FDA-approved test, that progressed on or after EGFR TKI therapy | 11/13/2015 |
| Atezolizumab (triple negative) | Unresectable locally advanced or metastatic triple-negative BC with tumors expressing PD-L1 (PD-L1-stained tumor-infiltrating immune cells of any intensity covering 1% of the tumor area), as determined by an FDA-approved test, in combination with paclitaxel protein-bound | 3/8/2019 |
| Palbociclib (ER+, HER2–) | ER+, HER2– mBC, in combination with letrozole for postmenopausal women as initial endocrine-based therapy for metastatic disease | 2/3/2015 |
| Fam-trastuzumab (HER2+) ≥3L | Unresectable or metastatic HER2+ BC in adults who have received two or more prior anti-HER2-based regimens in the metastatic setting | 12/20/2019 |
| Dabrafenib | Unresectable or metastatic melanoma with BRAF *V600E* or *V600K* mutations, as detected by an FDA-approved test, in combination with trametinib | 1/9/2014 |
| Nivolumab plus ipilimumab | BRAF *V600* wild-type unresectable or metastatic melanoma, in combination with ipilimumab | 9/30/2015 |
| Nivolumab (BRAF+) | - BRAF+ unresectable or metastatic melanoma, in combination with ipilimumab to remove the restriction for treatment of only patients with BRAF wild-type melanoma   BRAF *V600* mutation positive unresectable or metastatic melanoma, as a single agent, in order to remove the restriction that such patients should have disease progression following ipilimumab and a BRAF inhibitor | 1/23/2016 |
| Nivolumab ≥2L | Locally advanced or metastatic urothelial carcinoma that progressed:   - during or following platinum-containing chemotherapy - within 12 mo of neoadjuvant or adjuvant platinum-containing chemotherapy | 2/2/2017 |
| Pembrolizumab ≥2L | Unresectable or metastatic melanoma and disease progression following ipilimumab and, if BRAF *V600* mutation positive, a BRAF inhibitor | 9/4/2014 |
| Atezolizumab 1L | Locally mUC not eligible for cisplatin-containing chemotherapy and with tumors expressing PD-L1, as determined by an FDA-approved test, or not eligible for any platinum-containing chemotherapy regardless of PD-L1 status | 4/17/2017 |
| Pembrolizumab | mUC not eligible for cisplatin-containing chemotherapy | 5/18/2017 |
| Atezolizumab ≥2L | Locally advanced or metastatic urothelial carcinoma that progressed during or following platinum-containing chemotherapy or within 12 mo of neoadjuvant or adjuvant treatment with platinum-containing chemotherapy | 5/18/2016 |
| Erdafitinib ≥2L | mUC in adults that is susceptible FGFR3 or FGFR2 genetic alterations and has progressed during or following at least one line of prior platinum-containing chemotherapy, including within 12 mo of neoadjuvant or adjuvant platinum-containing chemotherapy; patients are selected for therapy based on an FDA-approved companion diagnostic for erdafitinib. | 4/12/2019 |
| Nivolumab ≥2L | Unresectable or metastatic melanoma and progression following ipilimumab and, if BRAF *V600* mutation positive, a BRAF inhibitor | 12/22/2014 |
| Enfortumab vedotin-ejfv ≥3L | mUC in adults who have received a PD-1 or PD-L1 inhibitor and a platinum-containing chemotherapy in the neoadjuvant, locally advanced, or metastatic setting | 12/18/2019 |
| Nivolumab ≥3L | Metastatic SCLC with progression after platinum-based chemotherapy and at least one other line of therapy | 8/16/2018 |

1L, first line; 2L, second line; 3L, third line; AA, accelerated approval; ALK, anaplastic lymphoma kinase; BRAF, v-raf murine sarcoma viral oncogene homolog B1; EGFR, epidermal growth factor receptor; ER, estrogen receptor; fam-trastuzumab, fam-trastuzumab deruxtecan-nxki; FDA, Food and Drug Administration; FGFR, fibroblast growth factor receptor; HER2, human epidermal growth factor receptor-2; mBC, metastatic breast cancer; mUC, advanced or metastatic urethral cancer; NSCLC, non-small cell lung cancer; PD-L1, programmed death ligand 1; SCLC, small cell lung cancer; TKI, tyrosine kinase inhibitor.
