# Supplementary material for: Real-World Clinical Oncology Outcomes Associated with the Accelerated Approval Pathway
Source: Cancer Res Commun. 2026 Jan 23;6(1):191–200. doi: 10.1158/2767-9764.CRC-25-0225 (PMC12828896; doi:10.1158/2767-9764.CRC-25-0225)
Supplement: Supplementary Table S2 — Table S2. Indication-specific inclusion criteria for analysis of outcomes of AA drugs in oncology solid tumors [file crc-25-0225_supplementary_table_s2_suppst2.docx]

**Supplementary Table S2.** Indication-specific inclusion criteria for analysis of outcomes of AA drugs in oncology solid tumors

| **Step** | **Arm** | **aNSCLC** | **N** | **Change** | **N (%)** |
| --- | --- | --- | --- | --- | --- |
| 1 |  | Advanced or metastatic NSCLC diagnosis from 1/1/11 to last datacut (May 2022, with data up to 4/30/22) | 77,398 |  |  |
| 2 |  | Treated with systemic therapy in Flatiron Health network within 120 days | 49,800 | 27,598 | 49800 (64.3) |
| 3 |  | Age ≥18 years | 49,232 | –568 | 49232 (63.6) |
|  |  | **ALK+ cohorts** |  |  |  |
| 4 |  | ALK+ cohort | 1,039 | –48,193 | 1039 (1.3) |
|  |  | ***Crizotinib analysis: Crizotinib (ALK+) vs controls (all-comers)*** |  |  |  |
|  | AA | Treatment with crizotinib between 8/26/11 to 11/20/13 whose ALK+ test was prior to crizotinib | 129 | –910 | 129 (0.2) |
|  | Control | Included all patients with NSCLC treatment that was not a product under AA between from 1/1/11 and 11/20/13 | 644 | –395 | 644 (0.8) |
|  |  | ***Ceritinib analysis Post-crizotinib)*** |  |  |  |
| 5 |  | Patients whose treatment after crizotinib occurred between 4/29/14 and 5/26/17 | 327 | –712 | 327 (0.4) |
|  | AA | First treatment after crizotinib was ceritinib (any) between 4/29/14 and 5/26/17 | 113 | –214 | 113 (0.1) |
|  | Control | First NSCLC treatment after crizotinib that was not a product under AA between 5/26/12 and 05/26/17 (excluded any other ALK inhibitor, ≥2L pembrolizumab between 10/02/15 and 10/24/16) | 52 | –275 | 52 (0.1) |
|  |  | ***Alectinib analysis (Post-ALK inhibitor)*** |  |  |  |
| 5 |  | Patients whose treatment after crizotinib or ceritinib occurred between 12/11/15 and 11/6/17 | 239 | –800 | 239 (0.3) |
|  | AA | Alectinib cohort 1 (AA): First treatment after first/second ALK inhibitor was alectinib between 12/11/15 and 11/6/17 | 118 | –121 | 118 (0.2) |
|  | Control | Alecitinib cohort (Historical): First treatment after first/second ALK inhibitor was any NSCLC treatment not under AA between 11/6/12 and 11/6/17 (excluded ceritinib until 5/27/17) and ≥2L pembrolizumab between 10/02/15 and 10/24/16 | 79 | –160 | 79 (0.1) |
|  |  | ***Brigatinib analysis (Post-ALK inhibitor)*** |  |  |  |
| 5 |  | Patients whose treatment after crizotinib, ceritinib, or alectinib occurred between 4/28/17 and 05/22/20 | 341 | –698 | 341 (0.4) |
|  | AA | Brigatinib cohort 1 (AA): First treatment after ALK inhibitor was brigatinib between 4/28/17 and 5/22/20 | 65 | –276 | 65 (0.1) |
|  | Control | Brigatinib cohort 3 (historical): First treatment after ALK inhibitor was any NSCLC treatment not under AA between 5/22/15 and 5/22/20 (excluded ceritinib before 5/27/17 and alectinib before 11/7/17) | 176 | –165 | 176 (0.2) |
|  |  | ***Lorlatinib analysis (Post-ALK inhibitor)*** |  |  |  |
| 5 |  | Patients whose treatment after crizotinib, ceritinib, clectinib, or brigatinib occurred between 11/2/18 and 3/3/21 | 201 | –838 | 201 (0.3) |
|  | AA | Lorlatinib cohort 1 (AA): Treatment after ALK inhibitor was lorlatinib between 11/2/18 and 3/3/21 | 78 | –123 | 78 (0.1) |
|  | Control | Lorlatinib cohort 3 (historical): Treatment after ALK inhibitor was any NSCLC treatment not under AA, between 3/3/16 and 3/3/21 (excluded brigatinib through 5/22/20) | 80 | –121 | 80 (0.1) |
|  |  | **CIT cohorts** |  |  |  |
| 4 |  | ***2L pembrolizumab analysis (PDL1+,*** *≥****2L)*** |  |  |  |
| 5 |  | Any PD-L1+ test | 20,488 | –28,744 | 20488 (26.5) |
|  | AA | ≥2L pembrolizumab cohort 1 (AA): Pembrolizumab in ≥2L between 10/02/15 and 10/24/16 and PD-L1+ before index treatment | 46 | –20,442 | 46 (0.1) |
|  | Control | ≥2L pembrolizumab cohort 2 (historical): Treated ≥2L chemotherapy between 1/1/11 and 10/24/16 or ≥2L nivolumab between 10/09/15 and 10/24/16 and PD-L1+ before index treatment | 68 | –20,420 | 68 (0.1) |
|  |  | ***1L pembrolizumab analysis (PDL1+, non-squamous, no driver mutation)*** |  |  |  |
|  |  | Non-squamous histology | 15,160 | –5,328 | 15160 (19.6) |
| 6 |  | No positive driver mutation (ALK, EGFR, ROS1, BRAF) | 9,634 | –5,526 | 9634 (12.4) |
|  | AA | 1L pembrolizumab cohort 1 (AA): Pembrolizumab, pemetrexed, carboplatin in 1L between 05/10/17 and 08/20/18 | 782 | –8,852 | 782 (1) |
|  | Control | 1L pembrolizumab cohort 2 (historical): Treated with 1L chemotherapy between 5/10/11 and 08/20/18 | 5,367 | –4,267 | 5367 (6.9) |
|  |  | **EGFR+ cohort** |  |  |  |
| 4 |  | EGFR+ test result (T790M) | 5,463 | –43,769 | 5463 (7.1) |
| 5 |  | Treated with EGFR TKI (erlotinib, gefitinib, afatinib) | 2,828 | –2,635 | 2828 (3.7) |
|  |  | ***Osimertinib analysis (EGFR+, post-EGFR TKI)*** |  |  |  |
|  | AA | Osimertinib cohort 1 (AA): Any first treatment after EGFR TKI between 11/13/15 and 03/30/17 | 220 | –2,608 | 220 (0.3) |
|  | Control | Osimertinib cohort 2 (historical): Any first treatment not including albociclib post-EGFR TKI between 1/1/11 and 3/30/17 | 633 | –2,195 | 633 (0.8) |
| **Step** | **Arm** | **mUC** | **N** | **Change** | **N (%)** |
| 1 |  | Patients with advanced urothelial diagnosis after 1/1/11 who was not in any other Flatiron Health datamart | 12,938 |  | 12,938 (100) |
| 2 |  | Treated within 120 days of advanced diagnosis | 7,485 | –5,453 | 7485 (57.9) |
| 3 |  | Aged ≥18 years at advanced diagnosis | 7,485 | 0 | 7485 (57.9) |
|  |  | **1L cis-ineligible** |  |  |  |
| 4 |  | Patients with PD-L1+ test result prior to 1L or who were not treated with carboplatin or cisplatin in 1L regardless of PD-L1 test | 2,814 | –4,671 | 2,814 (21.75) |
|  |  | ***Atezolizumab analysis (1L cis-ineligible)*** |  |  |  |
|  | AA | Treated with 1L atezolizumab between 4/17/17 and 12/2/22 | 427 | –2,387 | 427 (3.3) |
|  | Control | 1L treatment without atezolizumab between 4/17/12 and 12/2/22, and no pembrolizumab before 8/31/21 or avelumab before 6/30/20 | 763 | –2,051 | 763 (5.9) |
|  |  | ***Pembrolizumab analysis (1L cis-ineligible)*** |  |  |  |
|  | AA | Treated with 1L pembrolizumab between 5/18/17 and 8/31/21 | 812 | –2,002 | 812 (6.3) |
|  | Control | 1L treatment without pembrolizumab between 5/18/12 and 8/31/21, no avelumab before 6/30/20, and no atezolizumab at any time | 369 | –2,445 | 369 (2.9) |
|  |  | **≥2L post-platinum** |  |  |  |
| 4 |  | Treated with regimen containing carboplatin or cisplatin | 5,025 | –2,460 | 5025 (38.8) |
|  |  | ***Atezolizumab analysis (≥2L post-platinum)*** |  |  |  |
|  | AA | Atezolizumab in ≥2L between 5/18/16 and 4/13/21 and had cisplatin or carboplatin in prior line of therapy | 547 | –4,478 | 547 (4.2) |
|  | Control | Treated without atezolizumab in ≥2L between 5/18/16 and 4/13/21, with cisplatin or carboplatin in prior line of therapy, not treated with any CIT (other than pembrolizumab) except avelumab only after 6/30/20, and not treated with erdafitinib at any time | 1017 | –4,008 | 1017 (7.9) |
|  |  | ***Nivolumab analysis (≥2L post-platinum)*** |  |  |  |
|  | AA | Nivolumab in ≥2L between 2/2/17 and 8/19/21 and had cisplatin or carboplatin in prior line of therapy | 178 | –4,847 | 178 (1.4) |
|  | Control | Control: Treated without nivolumab in ≥2L between 2/2/12 and 8/19/21, had cisplatin or carboplatin in prior line of therapy, were not treated with any CIT (other than pembrolizumab) during AA period except avelumab only after 6/30/20, and were not treated with erdafetinib at any time during AA period | 1053 | –3,972 | 1053 (8.1) |
|  |  | ***Erdafitinib analysis (≥2L post-platinum, FGFR+)*** |  |  |  |
|  | AA | Erdafitinib in ≥2L between 4/12/19 and 5/31/2023 (end of data), had cisplatin or carboplatin in prior line of therapy, and were FGFR+ before treatment with erdafitinib or had specimen collected before treatment and the result date was within two weeks of the start date | 61 | –4,964 | 61 (0.5) |
|  | Control | Treated without erdafitinib in ≥2L between 4/12/14 and 5/31/2023 (end of data), had cisplatin or carboplatin in prior line of therapy, were not treated with any CIT (other than pembrolizumab) during AA period except avelumab only after 6/30/20 and nivolumab only after 8/19/21, and had a FGFR+ test before line of therapy start | 87 | –4,938 | 87 (0.7) |
|  |  | **≥3L post-platinum and post-CIT** |  |  |  |
| 4 |  | Treated with regimen containing carboplatin or cisplatin | 5,025 | –2,460 | 5025 (38.8) |
| 5 |  | Treated with a CIT | 2,224 | –2,801 | 2224 (17.2) |
|  |  | ***Enfortumab vedotin-ejfv analysis (≥3L post-platinum, post-CIT)*** |  |  |  |
|  | AA | Enfortumab vedotin-ejfv in ≥3L between 12/18/19 and 7/9/21, had cisplatin or carboplatin in a prior line of therapy, and a CIT in a prior line of therapy | 124 | –2,100 | 124 (1) |
|  | Control | Treated without enfortumab vedotin-ejfv ≥3L between 12/18/14 and 7/9/21, had cisplatin or carboplatin in prior line of therapy, had a CIT in a prior line of therapy, were not treated with any CIT (other than pembrolizumab) except evelumab only after 6/30/20, and were not treated with erdafetinib at any time | 223 | –2,001 | 223 (1.7) |
|  |  |  |  |  |  |
| **Step** | **Arm** | **Advanced or metastatic melanoma** | **N** | **Change** | **N (%)** |
| 1 |  | Advanced unresectable or metastatic melanoma diagnosis on or after 1/1/11; patient was not in any other Flatiron Health datamart | 10,819 |  | 10,819 (100) |
| 2 |  | Treated within 120 days of advanced diagnosis | 4,910 | –5,909 | 4910 (45.4) |
| 3 |  | Aged ≥18 years at advanced diagnosis | 4,909 | –1 | 4909 (45.4) |
|  |  | **1L** |  |  |  |
| 4 |  | 1L treatment is not a clinical study drug | 4,590 | –319 | 4,590 (42.43) |
|  |  | ***1L nivolumab plus ipilimumab, BRAF agnostic analysis*** |  |  |  |
|  | AA | Treated with 1L nivolumab plus ipilimumab between 9/30/15 and 3/7/19 | 478 | –4,112 | 478 (4.4) |
|  | Control | 1L treatment (without nivolumab) between 1/1/11 and 3/7/19, did not receive pembrolizumab before 12/18/15, and did not receive dabrafenib plus trametinib before 11/20/15 | 1517 | –3,073 | 1517 (14) |
|  |  | ***1L nivolumab monotherapy, BRAF+ analysis*** |  |  |  |
| 5 |  | BRAF+ test prior to 1L start | 1773 | –2,817 | 1773 (16.4) |
|  | AA | Treated with 1L nivolumab (monotherapy) between 1/23/16 and 3/7/19 | 84 | –1,689 | 84 (0.8) |
|  | Control | 1L treatment (without nivolumab) between 1/23/11 and 3/7/19, did not receive pembrolizumab before 12/18/15, and did not receive dabrafenib plus trametinib before 11/20/15 | 637 | –1,136 | 637 (5.9) |
|  |  | **≥2L post-ipilimumab** |  |  |  |
| 4 |  | Treated with ipilimumab | 2,510 | –2,399 | 2,510 (23.2) |
|  |  | ***≥2L nivolumab analysis (post-ipilimumab)*** |  |  |  |
|  | AA | Treated with nivolumab (monotherapy or combination therapy) any time after ipilimumab between 12/22/14 and 3/7/19 | 69 | –2,441 | 69 (0.6) |
|  | Control | ≥2L treatment (without nivolumab) any time after ipilimumab between 1/23/11 and 3/7/19, did not receive pembrolizumab before 12/18/15, and did not receive dabrafenib plus trametinib before 11/20/15 | 69 | –2,441 | 69 (0.6) |
|  |  | ***≥2L pembrolizumab analysis (post-ipilimumab)*** |  |  |  |
|  | AA | Treated with pembrolizumab any time after ipilimumab between 9/4/14 and 12/18/15 | 90 | –2,420 | 90 (0.8) |
|  | Control | ≥2L treatment (without pembrolizumab) any time after ipilimumab between 1/1/11 and 12/18/15, did not receive nivolumab before 3/7/19, and did not receive dabrafenib plus trametinib before 11/20/15 | 46 | –2,464 | 46 (0.4) |
|  |  | **BRAF+** |  |  |  |
| 4 |  | BRAF+ (any time) | 1,922 | –2,987 | 1,922 (17.77) |
|  |  | ***Dabrefenib plus trametinib analysis (BRAF+, any line of therapy)*** |  |  |  |
|  | AA | Treated with dabrafenib plus trametinib in any line any time after BRAF+ test between 1/9/14 and 11/20/15 | 158 | –1,764 | 158 (1.5) |
|  | Control | Treated in any line (without dabrafenib plus trametinib) after BRAF+ test between 1/1/11 and 11/20/15, did not receive pembrolizumab before 12/18/15, and did not receive nivolumab before 3/7/19 | 109 | –1,813 | 109 (1) |
| **Step** | **Arm** | **mBC** | **N** | **Change** | **N (%)** |
| 1 |  | mBC diagnosis on or after 1/1/11; patient was not in any other Flatiron Health datamart | 34,769 |  | 34,769 (100) |
| 2 |  | Treated within 120 days of initial diagnosis | 21,306 | –13,463 | 21306 (61.3) |
| 3 |  | Aged ≥18 years at initial diagnosis | 21,306 | 0 | 21306 (61.3) |
|  |  | ***1L palbociclib plus aromatase inhibitor analysis (ER+, HER2***–***)*** |  |  |  |
| 4 |  | ER+ and HER2– before 1L start (evidence of at least one ER+, evidence of HER2– and no HER2+) | 11741 | –9,565 | 11741 (33.8) |
|  | AA | Treated with 1L palbociclib plus aromatase inhibitor (anastrozole, letrozole, exemestane) with or without any other treatment between 2/3/15 and 3/31/17 | 551 | –11,190 | 551 (1.6) |
|  | Control | 1L treatment with a regimen that included one of the following (monotherapy or combination therapy): aromatase inhibitor (anastrozole, letrozole, exemestane), tamoxifen, or fulvestrant between 1/1/2011 and 3/31/17 | 2830 | –8,911 | 2830 (8.1) |
|  |  | ***≥3L HER2+ fam-trastuzumab deruxtecan-nxki (“enhertu”) analysis (HER2+)*** |  |  |  |
| 4 |  | HER2+ | 4241 | –17,065 | 4241 (12.2) |
|  | AA | Treated with enhertu after ≥2 other anti-HER2 lines of therapy (regimens containing at least one of the following: trastuzumab, pertuzumab, tucatinib, ado-trastuzumab emtansine, lapatinib, neratinib, margetuximab-cmkb) between 12/20/19 and 5/5/22 | 194 | –4,047 | 194 (0.6) |
|  | Control | Treatment after ≥2 other anti-HER2 lines of therapy between 12/20/14 and 5/5/22 | 418 | –3,823 | 418 (1.2) |
|  |  | ***1L atezolizumab plus paclitaxel analysis (Triple negative: ER***–***, PR***–***, HER2***–***)*** |  |  |  |
| 4 |  | ER–, PR–, and HER2– before 1L start (evidence of at least one negative test and no positive test) | 2144 | –19,162 | 2144 (6.2) |
|  | AA | Treated with atezolizumab plus paclitaxel between 03/08/19 and 10/6/21 | 148 | –1,996 | 148 (0.4) |
|  | Control | 1L treatment between 03/08/14 and 10/6/21 | 1279 | –865 | 1279 (3.7) |
| **Step** | **Arm** | **SCLC** | **N** | **Change** | **N ()** |
| 1 |  | SCLC diagnosis with extensive disease on or after 1/1/13; patient was not in any other Flatiron Health datamart | 6,453 |  | 6,453 (100) |
| 2 |  | Treated within 120 days of initial diagnosis | 5,076 | –1,377 | 5076 (78.7) |
| 3 |  | Aged ≥18 years at initial diagnosis | 5,076 | 0 | 5076 (78.7) |
| 4 |  | Treated with ≥2 lines of therapy, including a platinum-based therapy | 2,128 | –2,948 | 2128 (33) |
|  |  | ***≥3L nivolumab analysis (post-platinum)*** |  |  |  |
|  | AA | Treated with nivolumab after platinum therapy and at least one other line of therapy between 8/6/18 and 11/29/20 | 81 | –2,047 | 81 (1.3) |
|  | Control | ≥3L treatment (without nivolumab) after platinum therapy, at least one other line of therapy between 8/6/18 and 11/29/20, and did not receive without pembrolizumab before 3/30/21 | 273 | –1,855 | 273 (4.2) |

1L, first line; 2L, second line; 3L, third line; AA, accelerated approval; ALK, anaplastic lymphoma kinase; aNSCLC, advanced or metastatic non-small cell lung cancer; BRAF, v-raf murine sarcoma viral oncogene homolog B1; CIT, cancer immunotherapy; EGFR, epidermal growth factor receptor; ER, estrogen receptor; fam-trastuzumab, fam-trastuzumab deruxtecan-nxki; FGFR, fibroblast growth factor receptor; HER2, human epidermal growth factor receptor-2; mBC, metastatic breast cancer; mUC, advanced or metastatic urethral cancer; PD-L1, programmed death ligand 1; PR, progesterone receptor-negative; SCLC, small cell lung cancer; TKI, tyrosine kinase inhibitor.
