# Supplementary material for: Real-World Clinical Oncology Outcomes Associated with the Accelerated Approval Pathway
Source: Cancer Res Commun. 2026 Jan 23;6(1):191–200. doi: 10.1158/2767-9764.CRC-25-0225 (PMC12828896; doi:10.1158/2767-9764.CRC-25-0225)
Supplement: Supplementary Table S3 — Table S3. Additional detail on population impact extrapolation [file crc-25-0225_supplementary_table_s3_suppst3.docx]

## **Supplementary Table S3.** Additional detail on population impact extrapolation

| $Number of PFS/OS years gained at US population level=$ |
| --- |
| *Step 1:*  $\left[ \sum_{Start of AA Period}^{End of AA Period} \left( Number of treated patients across indicated LOTs \right) \right]\times\left( \% patients in indication treated with AA drug \right)$ |
| *Step 2:*  $\left[ Take sum from above \right]\times\left( {Survival}_{AA drug}-{Survival}_{SoC} \right)$ |

Number of treated patients across LOTs, CancerMPact 2024; % of patients in AA indication treated with AA drug, Flatiron Health.

AA, accelerated approval; LOT, line of therapy.

CancerMPact® utilizes a number of different sources in their calculation to estimate the number of treated patients across lines of therapy in the U.S. including population-based registries or databases such as U.S. Cancer Statistics and NODA (National Oncology Data Alliance), as well as survey-based data collected through their Treatment Architecture Survey (an internet survey of oncology providers)
