# Supplementary material for: Real-World Clinical Oncology Outcomes Associated with the Accelerated Approval Pathway
Source: Cancer Res Commun. 2026 Jan 23;6(1):191–200. doi: 10.1158/2767-9764.CRC-25-0225 (PMC12828896; doi:10.1158/2767-9764.CRC-25-0225)
Supplement: Supplementary Table S4 — Table S4. Baseline patient characteristics among those with aNSCLC [file crc-25-0225_supplementary_table_s4_suppst4.docx]

## **Supplementary Table S4.** Baseline patient characteristics among those with aNSCLC

| **Characteristic** | **Control (n=52)** | **Ceritinib (n = 113)** | ***P*** | **Control (n = 644)** | **Crizotinib (n = 129)** | ***P*** | **Control (n = 79)** | **Alectinib (n = 118)** | ***P*** | **Control (n = 176)** | **Brigatinib (n = 65)** | ***P*** | **Control (n = 80)** | **Lorlatinib (n = 78)** | ***P*** | **Control (n =5,367)** | **Pembrol-izumab 1L  (n = 782)** | ***P*** | **Control (n = 68)** | **Pembrol-izumab 2L  (n = 46)** | ***P*** | **Control (n = 633)** | **Osimert-inib  (n = 220)** | ***P*** |
| --- | --- | --- | --- | --- | --- | --- | --- | --- | --- | --- | --- | --- | --- | --- | --- | --- | --- | --- | --- | --- | --- | --- | --- | --- |
| **Age** |  |  | 0.499 |  |  | <0.001 |  |  | 0.62 |  |  | 0.283 |  |  | 0.34 |  |  | 0.903 |  |  | 0.885 |  |  | <0.001 |
| Mean (SD) | 59 (13) | 58 (13) |  | 67 (9) | 58 (12) |  | 59 (12) | 59 (12) |  | 61 (12) | 60 (12) |  | 63 (13) | 61 (11) |  | 67 (9) | 67 (9) |  | 66 (10) | 66 (10) |  | 67 (10) | 64 (11) |  |
| Median (IQR) | 62 (50 to 69) | 59 (49 to 66) |  | 69 (61 to 74) | 61 (50 to 68) |  | 62 (50 to 69) | 60 (52 to 67) |  | 64 (52 to 70) | 61 (53 to 67) |  | 64 (54 to 73) | 62 (55 to 68) |  | 68 (60 to 75) | 68 (60 to 74) |  | 64 (59 to 74) | 67 (60 to 74) |  | 69 (61 to 76) | 64 (56 to 73) |  |
| Range | 30 to 79 | 29 to 80 |  | 33 to 76 | 28 to 76 |  | 30 to 80 | 25 to 80 |  | 30 to 82 | 31 to 82 |  | 32 to 82 | 36 to 83 |  | 21 to 81 | 27 to 81 |  | 34 to 79 | 46 to 79 |  | 33 to 80 | 33 to 80 |  |
| **Histology, n (%)** |  |  | 0.042 |  |  | 0.997 |  |  | <0.001 |  |  | 0.332 |  |  | 0.027 |  |  |  |  |  | 0.474 |  |  | 0.092 |
| Non-squamous cell carcinoma | 44 (85) | 108 (96) |  | 609 (95) | 122 (95) |  | 69 (87) | 117 (99) |  | 164 (93) | 64 (98) |  | 80 (100) | 73 (94) |  | 5,367 (100) | 782 (100) |  | 55 (81) | 39 (85) |  | 610 (96) | 216 (98) |  |
| NSCLC histology NOS | 6 (12) | 3 (2.7) |  | 35 (5.4) | 7 (5.4) |  | 6 (7.6) | 0 (0) |  | 6 (3.4) | 0 (0) |  | 0 (0) | 1 (1.3) |  | - | - |  | 3 (4.4) | 0 (0) |  | 11 (1.7) | 4 (1.8) |  |
| Squamous cell carcinoma | 2 (3.8) | 2 (1.8) |  | - | - |  | 4 (5.1) | 1 (0.8) |  | 6 (3.4) | 1 (1.5) |  | 0 (0) | 4 (5.1) |  | - | - |  | 10 (15) | 7 (15) |  | 12 (1.9) | 0 (0) |  |
| **Sex, n (%)** |  |  | 0.56 |  |  | 0.377 |  |  | 0.428 |  |  | 0.908 |  |  | 0.851 |  |  | 0.007 |  |  | 0.118 |  |  | 0.577 |
| Female | 26 (50) | 62 (55) |  | 317 (49) | 69 (53) |  | 41 (52) | 68 (58) |  | 96 (55) | 36 (55) |  | 46 (57) | 46 (59) |  | 2,646 (49) | 345 (44) |  | 24 (35) | 23 (50) |  | 433 (68) | 146 (66) |  |
| Male | 26 (50) | 51 (45) |  | 327 (51) | 60 (47) |  | 38 (48) | 50 (42) |  | 80 (45) | 29 (45) |  | 34 (42) | 32 (41) |  | 2,721 (51) | 437 (56) |  | 44 (65) | 23 (50) |  | 200 (32) | 74 (34) |  |
| **Race/ ethnicity, n (%)** |  |  | 0.687 |  |  | 0.327 |  |  | 0.404 |  |  | 0.731 |  |  | 0.646 |  |  | 0.002 |  |  | 0.5 |  |  | 0.121 |
| Non-Hispanic White | 33 (63) | 68 (60) |  | 404 (63) | 75 (58) |  | 49 (62) | 80 (68) |  | 104 (59) | 40 (62) |  | 47 (59) | 43 (55) |  | 3,531 (66) | 471 (60) |  | 47 (69) | 29 (63) |  | 371 (59) | 142 (65) |  |
| Other race/ unknown | 19 (37) | 45 (40) |  | 240 (37) | 54 (42) |  | 30 (38) | 38 (32) |  | 72 (41) | 25 (38) |  | 33 (41) | 35 (45) |  | 1,836 (34) | 311 (40) |  | 21 (31) | 17 (37) |  | 262 (41) | 78 (35) |  |
| **Region, n (%)** |  |  | 0.613 |  |  | 0.592 |  |  | 0.164 |  |  | 0.526 |  |  | 0.019 |  |  | <0.001 |  |  | 0.899 |  |  | <0.001 |
| Midwest | 6 (12) | 13 (12) |  | 54 (8.4) | 13 (10) |  | 10 (13) | 20 (17) |  | 21 (12) | 11 (17) |  | 15 (19) | 7 (9.0) |  | 868 (16) | 106 (14) |  | 3 (4.4) | 4 (8.7) |  | 52 (8.2) | 15 (6.8) |  |
| Northeast | 9 (17) | 18 (16) |  | 100 (16) | 26 (20) |  | 12 (15) | 25 (21) |  | 35 (20) | 8 (12) |  | 10 (12) | 16 (21) |  | 861 (16) | 148 (19) |  | 18 (26) | 13 (28) |  | 123 (19) | 38 (17) |  |
| South | 18 (35) | 29 (26) |  | 263 (41) | 45 (35) |  | 27 (34) | 22 (19) |  | 53 (30) | 18 (28) |  | 19 (24) | 20 (26) |  | 2,014 (38) | 329 (42) |  | 32 (47) | 19 (41) |  | 193 (30) | 43 (20) |  |
| Unknown/ missing | 12 (23) | 27 (24) |  | 116 (18) | 24 (19) |  | 17 (22) | 31 (26) |  | 38 (22) | 14 (22) |  | 12 (15) | 23 (29) |  | 839 (16) | 87 (11) |  | 6 (8.8) | 4 (8.7) |  | 138 (22) | 79 (36) |  |
| West | 7 (13) | 26 (23) |  | 111 (17) | 21 (16) |  | 13 (16) | 20 (17) |  | 29 (16) | 14 (22) |  | 24 (30) | 12 (15) |  | 785 (15) | 112 (14) |  | 9 (13) | 6 (13) |  | 127 (20) | 45 (20) |  |
| **Smoking status, n (%)** |  |  | <0.001 |  |  | <0.001 |  |  | 0.033 |  |  | 0.228 |  |  | 0.109 |  |  | 0.171 |  |  | 0.712 |  |  | 0.743 |
| History of smoking | 35 (67) | 37 (33) |  | 528 (82) | 56 (43) |  | 42 (53) | 43 (36) |  | 83 (47) | 25 (38) |  | 25 (31) | 34 (44) |  | 4,846 (90) | 718 (92) |  | 64 (94) | 42 (91) |  | 282 (45) | 104 (47) |  |
| No history of smoking | 17 (33) | 76 (67) |  | 116 (18) | 73 (57) |  | 37 (47) | 74 (63) |  | 93 (53) | 40 (62) |  | 55 (69) | 44 (56) |  | 504 (9.4) | 64 (8.2) |  | 4 (5.9) | 4 (8.7) |  | 346 (55) | 115 (52) |  |
| Unknown/not documented | - | - |  | - | - |  | 0 (0) | 1 (0.8) |  | - | - |  | - | - |  | 17 (0.3) | 0 (0) |  | - | - |  | 5 (0.8) | 1 (0.5) |  |
| **Line of therapy, n (%)** |  |  | 0.003 |  |  | 0.991 |  |  | 0.133 |  |  | 0.109 |  |  | 0.652 |  |  |  |  |  | <0.001 |  |  | <0.001 |
| 1L |  |  |  | 424 (66) | 85 (66) |  | - | - |  | - | - |  | - | - |  | 5,367 (100) | 782 (100) |  | - | - |  |  |  |  |
| 2L | 18 (35) | 67 (59) |  | 220 (34)^a^ | 44 (34)^a^ |  | 27 (34) | 53 (45) |  | 77 (44) | 21 (32) |  | 28 (35) | 30 (38) |  | - | - |  | 61 (90) | 29 (63) |  | 463 (73) | 108 (49) |  |
| ≥3L | 34 (65) | 46 (41) |  | - | - |  | 52 (66) | 65 (55) |  | 99 (56) | 44 (68) |  | 52 (65) | 48 (62) |  | - | - |  | 7 (10) | 17 (37) |  | 170 (27) | 112 (51) |  |
| **Occurrence, n (%)** |  |  | 0.054 |  |  | 0.094 |  |  | 0.079 |  |  | 0.229 |  |  | 0.038 |  |  | <0.001 |  |  | 0.362 |  |  | 0.036 |
| *De novo* | 44 (85) | 107 (95) |  | 523 (81) | 111 (86) |  | 72 (91) | 99 (84) |  | 144 (82) | 59 (91) |  | 62 (78) | 70 (90) |  | 4,305 (80) | 703 (90) |  | 54 (79) | 39 (85) |  | 515 (81) | 190 (86) |  |
| Recurrent | 7 (13) | 6 (5.3) |  | 102 (16) | 18 (14) |  | 6 (7.6) | 19 (16) |  | 31 (18) | 6 (9.2) |  | 18 (22) | 8 (10) |  | 986 (18) | 71 (9.1) |  | 13 (19) | 5 (11) |  | 109 (17) | 24 (11) |  |
| Unknown | 1 (1.9) | 0 (0) |  | 19 (3.0) | 0 (0) |  | 1 (1.3) | 0 (0) |  | 1 (0.6) | 0 (0) |  | - | - |  | 76 (1.4) | 8 (1.0) |  | 1 (1.5) | 2 (4.3) |  | 9 (1.4) | 6 (2.7) |  |
| **Number of prior ALK inhibitor, n (%)** |  |  | 0.066 | 0 (0) | 0 (0) |  |  |  | 0.552 |  |  | <0.001 |  |  | 0.31 |  |  |  |  |  |  |  |  |  |
| 1 | 44 (85) | 107 (95) |  | - | - |  | 55 (70) | 78 (66) |  | 119 (68) | 27 (42) |  | 37 (46) | 36 (46) |  | N/A | N/A |  | N/A | N/A |  | N/A | N/A |  |
| 2 | 7 (13) | 4 (3.5) |  | - | - |  | 21 (27) | 29 (25) |  | 46 (26) | 19 (29) |  | 28 (35) | 27 (35) |  | N/A | N/A |  | N/A | N/A |  | N/A | N/A |  |
| 3 | 1 (1.9) | 2 (1.8) |  | - | - |  | 3 (3.8) | 10 (8.5) |  | 11 (6.2) | 14 (22) |  | 15 (19) | 10 (13) |  | N/A | N/A |  | N/A | N/A |  | N/A | N/A |  |
| 4 | - | - |  | - | - |  | 0 (0) | 1 (0.8) |  | 0 (0) | 4 (6.2) |  | 0 (0) | 3 (3.8) |  | N/A | N/A |  | N/A | N/A |  | N/A | N/A |  |
| 5 | - | - |  | - | - |  | - | - |  | - | - |  | 0 (0) | 1 (1.3) |  | N/A | N/A |  | N/A | N/A |  | N/A | N/A |  |
| 6 | - | - |  | - | - |  | - | - |  | 0 (0) | 1 (1.5) |  | 0 (0) | 1 (1.3) |  | N/A | N/A |  | N/A | N/A |  | N/A | N/A |  |

^a^Includes ≥2L.

1L, first line; 2L, second line; 3L, third line; ALK, anaplastic lymphoma kinase; aNSCLC, advanced non-small cell lung cancer; N/A, not applicable; NOS, not otherwise specified.
