# Supplementary material for: Real-World Clinical Oncology Outcomes Associated with the Accelerated Approval Pathway
Source: Cancer Res Commun. 2026 Jan 23;6(1):191–200. doi: 10.1158/2767-9764.CRC-25-0225 (PMC12828896; doi:10.1158/2767-9764.CRC-25-0225)
Supplement: Supplementary Table S5 — Table S5. Baseline patient characteristics among those with mBC [file crc-25-0225_supplementary_table_s5_suppst5.docx]

## **Supplementary Table S5.** Baseline patient characteristics among those with mBC

| **Characteristic** | **Control (n = 2,830)** | **1L palbociclib (ER+, HER2–) plus aromatase inhibitor (n = 551)** | ***P*** | **Control (n = 418)** | **Fam-trastuzumab (HER2+) ≥3L (n = 194)** | ***P*** | **Control (n = 1,279)** | **1L atezolizumab TNBC (n = 148)** | ***P*** |
| --- | --- | --- | --- | --- | --- | --- | --- | --- | --- |
| **Age** |  |  | <0.001 |  |  | 0.017 |  |  | 0.978 |
| Mean (SD) | 66.4 (12.4) | 63.8 (11.3) |  | 59.0 (13.4) | 61.4 (12.4) |  | 60.9 (13.1) | 60.9 (12.7) |  |
| Median (IQR) | 67.4 (58.5 to 76.7) | 65.0 (56.2 to 72.3) |  | 59.7 (50.5 to 67.9) | 63.5 (53.5 to 70.1) |  | 61.1 (51.7 to 71.0) | 62.0 (51.3 to 70.3) |  |
| Range | 24.5 to 86.4 | 29.6 to 85.2 |  | 28.1 to 85.2 | 30.7 to 85.2 |  | 22.5 to 85.5 | 36.4 to 85.3 |  |
| **Sex, n (%)** |  |  | 0.013 |  |  | 0.595 |  |  | 0.422 |
| Female | 2,788 (98.5) | 550 (99.8) |  | 416 (99.5) | 192 (99.0) |  | 1,275 (99.7) | 147 (99.3) |  |
| Male | 42 (1.5) | 1 (0.2) |  | 2 (0.5) | 2 (1.0) |  | 4 (0.3) | 1 (0.7) |  |
| **Race/ethnicity, n (%)** |  |  | 0.117 |  |  | 0.386 |  |  | 0.456 |
| Hispanic or Latino | 158 (5.6) | 24 (4.4) |  | 56 (13.4) | 17 (8.8) |  | 125 (9.8) | 14 (9.5) |  |
| Non-Hispanic Black/African American | 208 (7.3) | 31 (5.6) |  | 42 (10.0) | 23 (11.9) |  | 249 (19.5) | 22 (14.9) |  |
| Non-Hispanic White | 1,825 (64.5) | 383 (69.5) |  | 225 (53.8) | 106 (54.6) |  | 584 (45.7) | 68 (45.9) |  |
| Other/unknown | 639 (22.6) | 113 (20.5) |  | 95 (22.7) | 48 (24.7) |  | 321 (25.1) | 44 (29.7) |  |
| **Region, n (%)** |  |  | 0.845 |  |  | 0.977 |  |  | 0.151 |
| Midwest | 389 (16.8) | 73 (17.3) |  | 42 (13.8) | 22 (13.6) |  | 147 (15.0) | 13 (11.8) |  |
| Northeast | 495 (21.4) | 86 (20.4) |  | 54 (17.7) | 29 (17.9) |  | 161 (16.4) | 12 (10.9) |  |
| South | 972 (42.0) | 185 (43.8) |  | 136 (44.6) | 75 (46.3) |  | 522 (53.3) | 61 (55.5) |  |
| West | 458 (19.8) | 78 (18.5) |  | 73 (23.9) | 36 (22.2) |  | 150 (15.3) | 24 (21.8) |  |
| Missing | 516 | 129 |  | 113 | 32 |  | 299 | 38 |  |
| **Stage at initial diagnosis, n (%)** |  |  | 0.152 |  |  | 0.371 |  |  | 0.125 |
| I | 341 (12.0) | 66 (12.0) |  | 27 (6.5) | 18 (9.3) |  | 129 (10.1) | 8 (5.4) |  |
| II | 681 (24.1) | 136 (24.7) |  | 80 (19.1) | 38 (19.6) |  | 315 (24.6) | 40 (27.0) |  |
| III | 481 (17.0) | 83 (15.1) |  | 77 (18.4) | 26 (13.4) |  | 314 (24.6) | 36 (24.3) |  |
| IV | 1,006 (35.5) | 219 (39.7) |  | 213 (51.0) | 105 (54.1) |  | 433 (33.9) | 59 (39.9) |  |
| Not documented | 321 (11.3) | 47 (8.5) |  | 21 (5.0) | 7 (3.6) |  | 88 (6.9) | 5 (3.4) |  |
| **ECOG, n (%)** |  |  | <0.001 |  |  | <0.001 |  |  | 0.03 |
| 0 | 507 (17.9) | 146 (26.5) |  | 114 (27.3) | 52 (26.8) |  | 405 (31.7) | 58 (39.2) |  |
| 1 | 424 (15.0) | 105 (19.1) |  | 160 (38.3) | 78 (40.2) |  | 315 (24.6) | 43 (29.1) |  |
| ≥2 | 242 (8.6) | 32 (5.8) |  | 40 (9.6) | 37 (19.1) |  | 139 (10.9) | 8 (5.4) |  |
| Not documented | 1,657 (58.6) | 268 (48.6) |  | 104 (24.9) | 27 (13.9) |  | 420 (32.8) | 39 (26.4) |  |
| **Line of therapy, n (%)** |  |  |  |  |  | 0.99 |  |  |  |
| 1L | 2,830 (100) | 551 (100) |  |  |  |  | 1,279 (100) | 148 (100) |  |
| 2L |  |  |  |  |  |  |  |  |  |
| 3L |  |  |  | 86 (20.6) | 40 (20.6) |  |  |  |  |
| ≥4L |  |  |  | 332 (79.4) | 154 (79.4) |  |  |  |  |

1L, first line; 2L, second line; 3L, third line; 4L, fourth line; ECOG, Eastern Cooperative Oncology Group; ER, estrogen receptor; HER2, human epidermal growth factor receptor 2; IQR, interquartile range; mBC, metastatic breast cancer; SD, standard deviation; TNBC, triple negative breast cancer.
