# Supplementary material for: Real-World Clinical Oncology Outcomes Associated with the Accelerated Approval Pathway
Source: Cancer Res Commun. 2026 Jan 23;6(1):191–200. doi: 10.1158/2767-9764.CRC-25-0225 (PMC12828896; doi:10.1158/2767-9764.CRC-25-0225)
Supplement: Supplementary Table S6 — Table S6. Baseline Patient Characteristics Among Those With Melanoma [file crc-25-0225_supplementary_table_s6_suppst6.docx]

## **Supplementary Table S6.** Baseline Patient Characteristics Among Those With Melanoma

| **Characteristic** | **Control**, (N=637) | **Nivolumab monotherapy 1L, BRAF+** (n=84) | ***P*** | **Control**, (n=1,517) | **Nivolumab plus ipilimumab 1L, BRAF agnostic** (n=478) | ***P*** | **Control**, (n=69) | **Nivolumab post-ipilimumab** (n=69) | ***P*** | **Control** (n=46) | **Pembrolizumab post-ipilimumab** (n=90) | ***P*** | **Control**, (n=109) | **Dabrafenib (plus trametinib) ≥1L** (n=158) | ***P*** |
| --- | --- | --- | --- | --- | --- | --- | --- | --- | --- | --- | --- | --- | --- | --- | --- |
| **Age** |  |  | 0.106 |  |  | <0*.*001 |  |  | 0.515 |  |  | *.*971 |  |  | *.*609 |
| Mean (SD) | 61.8 (13.7) | 64.1 (13.4) |  | 66.4 (13.3) | 62.6 (13.4) |  | 64.9 (12.8) | 66.0 (13.4) |  | 65.2 (13.1) | 64.9 (13.3) |  | 57.3 (14.7) | 58.3 (14.4) |  |
| Median (IQR) | 62.6 (53.1 to 72.3) | 65.8 (55.7 to 75.0) |  | 68.0 (58.1 to 77.1) | 64.5 (55.1 to 71.9) |  | 67.3 (58.0 to 75.4) | 69.1 (55.3 to 77.1) |  | 67.4 (57.8 to 76.0) | 67.5 (57.8 to 75.5) |  | 58.7 (48.5 to 66.1) | 59.8 (50.3 to 68.2) |  |
| Range | 18.7 to 85.4 | 32.2 to 84.9 |  | 18.7 to 85.4 | 19.7 to 85.5 |  | 27.4 to 84.4 | 31.4 to 84.4 |  | 27.4 to 84.4 | 29.2 to 84.8 |  | 19.1 to 84.4 | 19.2 to 85.3 |  |
| **Sex, n (%)** |  |  | .831 |  |  | 0.205 |  |  | 0.59 |  |  | 0.64 |  |  | 0.683 |
| Female | 220 (34.5) | 30 (35.7) |  | 488 (32.2) | 139 (29.1) |  | 25 (36.2) | 22 (31.9) |  | 16 (34.8) | 35 (38.9) |  | 42 (38.5) | 57 (36.1) |  |
| Male | 417 (65.5) | 54 (64.3) |  | 1,029 (67.8) | 339 (70.9) |  | 44 (63.8) | 47 (68.1) |  | 30 (65.2) | 55 (61.1) |  | 67 (61.5) | 101 (63.9) |  |
| **Race/ethnicity, n (%)** |  |  | 0.103 |  |  | 0.187 |  |  | >0.999 |  |  | 0.506 |  |  | 0.088 |
| Hispanic or Latino | 18 (2.8) | 2 (2.4) |  | 38 (2.5) | 10 (2.1) |  | 2 (2.9) | 1 (1.4) |  | 1 (2.2) | 2 (2.2) |  | 6 (5.5) | 2 (1.3) |  |
| Non-Hispanic Black/African American | 2 (0.3) | 0 (0.0) |  | 4 (0.3) | 5 (1.0) |  | 1 (1.4) | 0 (0.0) |  | 0 (0.0) | 1 (1.1) |  | 1 (0.9) | 0 (0.0) |  |
| Non-Hispanic White | 493 (77.4) | 56 (66.7) |  | 1,147 (75.6) | 361 (75.5) |  | 50 (72.5) | 51 (73.9) |  | 33 (71.7) | 72 (80.0) |  | 83 (76.1) | 132 (83.5) |  |
| Other/unknown | 124 (19.5) | 26 (31.0) |  | 328 (21.6) | 102 (21.3) |  | 16 (23.2) | 17 (24.6) |  | 12 (26.1) | 15 (16.7) |  | 19 (17.4) | 24 (15.2) |  |
| **Region, n (%)** |  |  | 0.689 |  |  | 0.102 |  |  | 0.621 |  |  | 0.708 |  |  | 0.488 |
| Midwest | 60 (12.9) | 13 (18.1) |  | 145 (13.4) | 57 (15.4) |  | 8 (15.1) | 7 (11.9) |  | 5 (13.2) | 8 (14.3) |  | 9 (11.0) | 17 (17.0) |  |
| Northeast | 63 (13.6) | 9 (12.5) |  | 147 (13.5) | 64 (17.3) |  | 8 (15.1) | 8 (13.6) |  | 7 (18.4) | 9 (16.1) |  | 19 (23.2) | 19 (19.0) |  |
| South | 232 (50.0) | 35 (48.6) |  | 511 (47.1) | 172 (46.4) |  | 25 (47.2) | 24 (40.7) |  | 19 (50.0) | 23 (41.1) |  | 30 (36.6) | 41 (41.0) |  |
| West | 109 (23.5) | 15 (20.8) |  | 282 (26.0) | 78 (21.0) |  | 12 (22.6) | 20 (33.9) |  | 7 (18.4) | 16 (28.6) |  | 24 (29.3) | 23 (23.0) |  |
| Missing | 173 | 12 |  | 432 | 107 |  | 16 | 10 |  | 8 | 34 |  | 27 | 58 |  |
| **Stage at initial diagnosis, n (%)** |  |  | 0.546 |  |  |  |  |  | 0.95 |  |  | 0.287 |  |  | 0.022 |
| 0 | 1 (0.2) | 0 (0.0) |  | 5 (0.3) | 2 (0.4) |  | 5 (7.2) | 3 (4.3) |  | 0 (0.0) | 0 (0.0) |  | 1 (0.9) | 0 (0.0) |  |
| I | 56 (8.8) | 10 (11.9) |  | 136 (9.0) | 44 (9.2) |  | 20 (29.0) | 19 (27.5) |  | 2 (4.3) | 12 (13.3) |  | 6 (5.5) | 20 (12.7) |  |
| II | 106 (16.6) | 13 (15.5) |  | 301 (19.8) | 92 (19.2) |  | 1 (1.4) | 2 (2.9) |  | 11 (23.9) | 12 (13.3) |  | 16 (14.7) | 28 (17.7) |  |
| III | 30 (4.7) | 6 (7.1) |  | 60 (4.0) | 10 (2.1) |  | 28 (40.6) | 29 (42.0) |  | 1 (2.2) | 3 (3.3) |  | 7 (6.4) | 1 (0.6) |  |
| IV | 272 (42.7) | 38 (45.2) |  | 610 (40.2) | 217 (45.4) |  | 15 (21.7) | 16 (23.2) |  | 20 (43.5) | 35 (38.9) |  | 50 (45.9) | 68 (43.0) |  |
| Not documented | 172 (27.0) | 17 (20.2) |  | 405 (26.7) | 113 (23.6) |  |  |  | 0.604 | 12 (26.1) | 28 (31.1) |  | 29 (26.6) | 41 (25.9) |  |
| **ECOG, n (%)** |  |  | 0.004 |  |  | <0.001 | 10 (14.5) | 15 (21.7) |  |  |  | 0.051 |  |  | <0.001 |
| 0 | 137 (21.5) | 27 (32.1) |  | 321 (21.2) | 178 (37.2) |  | 18 (26.1) | 17 (24.6) |  | 4 (8.7) | 16 (17.8) |  | 8 (7.3) | 35 (22.2) |  |
| 1 | 140 (22.0) | 15 (17.9) |  | 331 (21.8) | 113 (23.6) |  | 9 (13.0) | 11 (15.9) |  | 10 (21.7) | 34 (37.8) |  | 22 (20.2) | 40 (25.3) |  |
| ≥2 | 66 (10.4) | 16 (19.0) |  | 156 (10.3) | 36 (7.5) |  | 32 (46.4) | 26 (37.7) |  | 8 (17.4) | 10 (11.1) |  | 9 (8.3) | 17 (10.8) |  |
| Not documented | 294 (46.2) | 26 (31.0) |  | 709 (46.7) | 151 (31.6) |  |  |  |  | 24 (52.2) | 30 (33.3) |  | 70 (64.2) | 66 (41.8) |  |
| **Line of therapy, n (%)** |  |  |  |  |  |  |  |  | <0.001 |  |  | <0*.*001 |  |  | 0.998 |
| 1L | 637 (100) | 84 (100) |  | 1,517 (100) | 478 (100) |  | - | - |  | - | - |  | 68 (62.4) | 98 (62.0) |  |
| 2L | - | - |  | - | - |  | 64 (92.8) | 34 (49.3) |  | 43 (93.5) | 57 (63.3) |  | 30 (27.5) | 44 (27.8) |  |
| ≥3L | - | - |  | - | - |  | 5 (7.2) | 35 (50.7) |  | 3 (6.5) | 33 (36.7) |  | 11 (10.1) | 16 (10.1) |  |

1L, first line; 2L, second line; 3L, third line; ECOG, Eastern Cooperative Oncology Group; IQR, interquartile range; SD, standard deviation.
