# Supplementary material for: Real-World Clinical Oncology Outcomes Associated with the Accelerated Approval Pathway
Source: Cancer Res Commun. 2026 Jan 23;6(1):191–200. doi: 10.1158/2767-9764.CRC-25-0225 (PMC12828896; doi:10.1158/2767-9764.CRC-25-0225)
Supplement: Supplementary Table S7 — Table S7. Baseline patient characteristics among those with mUC [file crc-25-0225_supplementary_table_s7_suppst7.docx]

## **Supplementary Table S7.** Baseline patient characteristics among those with mUC

| **Characteristic** | **Control  (n=763)** | **Atezolizumab 1L  (n=427)** | ***P*** | **Control (n=369)** | **Pembrolizumab 1L  (n=812)** | ***P*** | **Control (n=1,017)** | **Atezolizumab 2L+, post platinum (n=547)** | ***P*** | **Control (n=1,053)** | **Nivolumab ≥2L, post platinum (n=178)** | ***P*** | **Control (n=87)** | **Erdafitinib ≥2, FGFR+ (n=61)** | ***P*** | **Control (n=223)** | **Enfortumab ≥3L, post-platinumb and post-CIT  (n=124)** | ***P*** |
| --- | --- | --- | --- | --- | --- | --- | --- | --- | --- | --- | --- | --- | --- | --- | --- | --- | --- | --- |
| **Age** |  |  | <0.001 |  |  | <0.001 |  |  | 0.205 |  |  | 0.667 |  |  | 0.664 |  |  | 0.015 |
| Mean (SD) | 74.5 (8.8) | 76.7 (7.9) |  | 73.8 (9.0) | 75.6 (8.5) |  | 70.2 (9.9) | 71.1 (8.7) |  | 70.4 (9.7) | 70.9 (9.2) |  | 69.5 (10.9) | 70.2 (10.0) |  | 72.0 (8.7) | 69.1 (9.5) |  |
| Median (IQR) | 75.8 (69.2 to 82.3) | 79.1 (72.4 to 83.2) |  | 74.9 (68.5 to 81.5) | 77.8 (70.3 to 82.7) |  | 71.2 (63.7 to 78.1) | 71.7 (65.5 to 78.0) |  | 71.6 (64.2 to 78.2) | 71.1 (64.4 to 77.8) |  | 70.1 (64.3 to 77.3) | 72.4 (63.3 to 77.7) |  | 72.5 (65.8 to 79.5) | 70.0 (63.8 to 75.5) |  |
| Range | 39.4 to 85.5 | 33.9 to 85.4 |  | 39.4 to 85.3 | 40.4 to 85.4 |  | 29.0 to 85.4 | 34.7 to 85.3 |  | 29.0 to 85.4 | 32.2 to 85.4 |  | 24.7 to 85.0 | 43.2 to 85.3 |  | 51.7 to 85.1 | 35.9 to 85.0 |  |
| **Sex, n (%)** |  |  | 0.328 |  |  | 0.196 |  |  | 0.429 |  |  | 0.762 |  |  | 0.162 |  |  | 0.375 |
| Female | 198 (26.0) | 122 (28.6) |  | 89 (24.1) | 225 (27.7) |  | 283 (27.8) | 142 (26.0) |  | 296 (28.1) | 52 (29.2) |  | 26 (29.9) | 25 (41.0) |  | 55 (24.7) | 36 (29.0) |  |
| Male | 565 (74.0) | 305 (71.4) |  | 280 (75.9) | 587 (72.3) |  | 734 (72.2) | 405 (74.0) |  | 757 (71.9) | 126 (70.8) |  | 61 (70.1) | 36 (59.0) |  | 168 (75.3) | 88 (71.0) |  |
| **Race/ethnicity, n (%)** |  |  | 0.051 |  |  | 0.031 |  |  | 0.413 |  |  | 0.062 |  |  | 0.028 |  |  | 0.792 |
| Hispanic or Latino | 25 (3.3) | 17 (4.0) |  | 11 (3.0) | 19 (2.3) |  | 42 (4.1) | 20 (3.7) |  | 44 (4.2) | 15 (8.4) |  | 3 (3.4) | 1 (1.6) |  | 4 (1.8) | 2 (1.6) |  |
| Non-Hispanic Black/African American | 39 (5.1) | 18 (4.2) |  | 22 (6.0) | 24 (3.0) |  | 50 (4.9) | 20 (3.7) |  | 52 (4.9) | 5 (2.8) |  | 2 (2.3) | 2 (3.3) |  | 8 (3.6) | 5 (4.0) |  |
| Non-Hispanic White | 459 (60.2) | 226 (52.9) |  | 239 (64.8) | 512 (63.1) |  | 632 (62.1) | 361 (66.0) |  | 659 (62.6) | 108 (60.7) |  | 39 (44.8) | 41 (67.2) |  | 151 (67.7) | 78 (62.9) |  |
| Other/unknown | 240 (31.5) | 166 (38.9) |  | 97 (26.3) | 257 (31.7) |  | 293 (28.8) | 146 (26.7) |  | 298 (28.3) | 50 (28.1) |  | 43 (49.4) | 17 (27.9) |  | 60 (26.9) | 39 (31.5) |  |
| **Region, n (%)** |  |  | 0.324 |  |  | 0.219 |  |  | 0.439 |  |  | <0.001 |  |  | 0.033 |  |  | 0.039 |
| Midwest | 73 (12.4) | 46 (12.4) |  | 36 (12.5) | 97 (16.2) |  | 112 (11.0) | 69 (12.6) |  | 116 (11.0) | 30 (16.9) |  | 4 (4.6) | 6 (9.8) |  | 28 (12.6) | 11 (8.9) |  |
| Northeast | 90 (15.3) | 69 (18.6) |  | 36 (12.5) | 93 (15.5) |  | 127 (12.5) | 74 (13.5) |  | 132 (12.5) | 10 (5.6) |  | 12 (13.8) | 8 (13.1) |  | 39 (17.5) | 12 (9.7) |  |
| South | 342 (58.0) | 194 (52.4) |  | 170 (58.8) | 317 (52.9) |  | 399 (39.2) | 210 (38.4) |  | 408 (38.7) | 90 (50.6) |  | 49 (56.3) | 24 (39.3) |  | 78 (35.0) | 48 (38.7) |  |
| West | 85 (14.4) | 61 (16.5) |  | 47 (16.3) | 92 (15.4) |  | 253 (24.9) | 117 (21.4) |  | 261 (24.8) | 34 (19.1) |  | 9 (10.3) | 17 (27.9) |  | 49 (22.0) | 42 (33.9) |  |
| Missing | 173 | 57 |  | 80 | 213 |  | 126 (12.4) | 77 (14.1) |  | 136 (12.9) | 14 (7.9) |  | 13 (14.9) | 6 (9.8) |  | 29 (13.0) | 11 (8.9) |  |
| **Primary site, n (%)** |  |  | 0.344 |  |  | 0.451 |  |  | 0.625 |  |  | 0.757 |  |  | 0.799 |  |  | 0.495 |
| Bladder | 600 (78.6) | 329 (77.0) |  | 289 (78.3) | 615 (75.7) |  | 755 (74.2) | 394 (72.0) |  | 785 (74.5) | 128 (71.9) |  | 57 (65.5) | 39 (63.9) |  | 144 (64.6) | 73 (58.9) |  |
| Renal pelvis | 95 (12.5) | 49 (11.5) |  | 47 (12.7) | 105 (12.9) |  | 165 (16.2) | 98 (17.9) |  | 165 (15.7) | 31 (17.4) |  | 19 (21.8) | 12 (19.7) |  | 49 (22.0) | 34 (27.4) |  |
| Ureter/urethra | 68 (8.9) | 49 (11.5) |  | 33 (8.9) | 92 (11.3) |  | 97 (9.5) | 55 (10.1) |  | 103 (9.8) | 19 (10.7) |  | 11 (12.6) | 10 (16.4) |  | 30 (13.5) | 17 (13.7) |  |
| **ECOG, n (%)** |  |  | <0.001 |  |  | <0.001 |  |  | 0.003 |  |  | 0.03 |  |  | 0.08 |  |  | 0.944 |
| 0 | 144 (18.9) | 84 (19.7) |  | 52 (14.1) | 167 (20.6) |  | 178 (17.5) | 110 (20.1) |  | 193 (18.3) | 35 (19.7) |  | 23 (26.4) | 15 (24.6) |  | 44 (19.7) | 23 (18.5) |  |
| 1 | 227 (29.8) | 146 (34.2) |  | 93 (25.2) | 273 (33.6) |  | 382 (37.6) | 214 (39.1) |  | 399 (37.9) | 73 (41.0) |  | 43 (49.4) | 25 (41.0) |  | 88 (39.5) | 49 (39.5) |  |
| ≥2 | 153 (20.1) | 114 (26.7) |  | 70 (19.0) | 174 (21.4) |  | 145 (14.3) | 100 (18.3) |  | 155 (14.7) | 36 (20.2) |  | 14 (16.1) | 7 (11.5) |  | 55 (24.7) | 29 (23.4) |  |
| Not documented | 239 (31.3) | 83 (19.4) |  | 154 (41.7) | 198 (24.4) |  | 312 (30.7) | 123 (22.5) |  | 306 (29.1) | 34 (19.1) |  | 7 (8.0) | 14 (23.0) |  | 36 (16.1) | 23 (18.5) |  |
| **Line of therapy, n (%)** |  |  |  |  |  |  |  |  | <0.001 |  |  | <0.001 |  |  | <0.001 |  |  | 0.981 |
| 1L | 763 (100) | 427 (100) |  | 369 (100) | 812 (100) |  | - | - |  | - | - |  | - | - |  | - | - |  |
| 2L | - | - |  | - | - |  | 979 (96.3) | 419 (76.6) |  | 1,003 (95.3) | 130 (73.0) |  | 79 (90.8) | 20 (32.8) |  | - | - |  |
| ≥3L | - | - |  | - | - |  | 38 (3.7)^a^ | 128 (23.4)^a^ |  | 50 (4.7)^a^ | 48 (27.0)^a^ |  | 8 (9.2)^a^ | 41 (67.2)^a^ |  | 122 (54.7) | 68 (54.8) |  |
| ≥4L | - | - |  | - | - |  | N/A | N/A |  | N/A | N/A |  | N/A | N/A |  | 101 (45.3) | 56 (45.2) |  |

^a^Includes ≥3L.

1L, first line; 2L, second line; 3L, third line; CIT, cancer immunotherapy; ECOG, Eastern Cooperative Oncology Group; FGFR, fibroblast growth factor receptors; IQR, interquartile range; mUC, metastatic urothelial carcinoma; N/A, not applicable; SD, standard deviation.
