# Supplementary material for: Real-World Clinical Oncology Outcomes Associated with the Accelerated Approval Pathway
Source: Cancer Res Commun. 2026 Jan 23;6(1):191–200. doi: 10.1158/2767-9764.CRC-25-0225 (PMC12828896; doi:10.1158/2767-9764.CRC-25-0225)
Supplement: Supplementary Table S8 — Table S8. Baseline patient characteristics among those with SCLC [file crc-25-0225_supplementary_table_s8_suppst8.docx]

## **Supplementary Table S8.** Baseline patient characteristics among those with SCLC

| **Characteristic** | **Control (n = 302)** | **Nivolumab ≥3L (n = 81)** | ***P*** |
| --- | --- | --- | --- |
| **Age** |  |  | 0.27 |
| Mean (SD) | 65.9 (8.8) | 64.7 (8.1) |  |
| Median (IQR) | 66.0 (59.9 to 72.1) | 64.9 (60.1 to 69.7) |  |
| Range | 41.5 to 85.3 | 41.8 to 85.1 |  |
| **Sex, n (%)** |  |  | 0.677 |
| Female | 145 (48.0) | 41 (50.6) |  |
| Male | 157 (52.0) | 40 (49.4) |  |
| **Race/ethnicity, n (%)** |  |  | 0.598 |
| Hispanic or Latino | 7 (2.3) | 1 (1.2) |  |
| Non-Hispanic Black/African American | 19 (6.3) | 4 (4.9) |  |
| Non-Hispanic White | 202 (66.9) | 50 (61.7) |  |
| Other/unknown | 74 (24.5) | 26 (32.1) |  |
| **Region, n (%)** |  |  | 0.018 |
| Midwest | 46 (19.6) | 9 (13.4) |  |
| Northeast | 54 (23.0) | 6 (9.0) |  |
| South | 104 (44.3) | 42 (62.7) |  |
| West | 31 (13.2) | 10 (14.9) |  |
| Missing | 67 | 14 |  |
| **Stage at initial diagnosis, n (%)** |  |  | 0.852 |
| III | 1 (0.3) | 0 (0.0) |  |
| IV | 278 (92.1) | 76 (93.8) |  |
| Unknown/not documented | 23 (7.6) | 5 (6.2) |  |
| **ECOG, n (%)** |  |  | 0.028 |
| 0 | 33 (10.9) | 14 (17.3) |  |
| 1 | 99 (32.8) | 33 (40.7) |  |
| ≥2 | 73 (24.2) | 21 (25.9) |  |
| Not documented | 97 (32.1) | 13 (16.0) |  |
| **Line of therapy, n (%)** |  |  | 0.992 |
| 3L | 231 (76.5) | 62 (76.5) |  |
| ≥4L | 71 (23.5) | 19 (23.5) |  |

3L, third line; 4L, fourth line; ECOG, Eastern Cooperative Oncology Group; IQR, interquartile range; SCLC, small cell lung cancer; SD, standard deviation.**Supplementary Table S9.** Unweighted risk of progression or death and risk of death for AA drugs vs SoC

|  |  |  |  | **rwPFS** |  |  |  | **OS** |  |
| --- | --- | --- | --- | --- | --- | --- | --- | --- | --- |
|  |  |  | **HR** | **95% CI** | ***P*** |  | **HR** | **95% CI** | ***P*** |
| aNSCLC | ALK+ | Alectinib ≥2L | 0.32 | 0.23 to 0.46 | <0.001 |  | 0.28 | 0.20 to 0.40 | <0.001 |
|  |  | Brigatinib ≥2L | 0.89 | 0.63 to 1.26 | 0.5 |  | 0.68 | 0.48 to 0.97 | 0.035 |
|  |  | Ceritinib ≥2L | 0.41 | 0.28 to 0.62 | <0.001 |  | 0.44 | 0.30 to 0.63 | <0.001 |
|  |  | Crizotinib | 0.6 | 0.48 to 0.75 | <0.001 |  | 0.4 | 0.32 to 0.50 | <0.001 |
|  |  | Lorlatinib ≥2L | 0.83 | 0.56 to 1.21 | 0.3 |  | 0.77 | 0.52 to 1.14 | 0.2 |
|  | CIT | Pembrolizumab 1L | 0.8 | 0.73 to 0.88 | <0.001 |  | 0.99 | 0.91 to 1.09 | >0.9 |
|  |  | Pembrolizumab ≥2L | 0.89 | 0.58 to 1.35 | 0.6 |  | 1 | 0.66 to 1.53 | >0.9 |
|  | EGFR+ | Osimertinib | 0.66 | 0.55 to 0.78 | <0.001 |  | 0.66 | 0.56 to 0.78 | <0.001 |
| mBC | 1L | Atezolizumab (triple negative) | 0.8 | 0.66 to 0.98 | 0.029 |  | 0.73 | 0.59 to 0.91 | 0.004 |
|  |  | Palbociclib (ER+, HER2–) | 0.73 | 0.65 to 0.82 | <0.001 |  | 0.77 | 0.69 to 0.87 | <0.001 |
|  | ≥3L | Fam-trastuzumab (HER2+) ≥3L | 0.61 | 0.49 to 0.76 | <0.001 |  | 0.77 | 0.61 to 0.97 | 0.027 |
| Melanoma | BRAF+ | Dabrafenib | 0.97 | 0.73 to 1.28 | 0.8 |  | 0.96 | 0.72 to 1.27 | 0.8 |
|  | 1L | Nivolumab plus ipilimumab | 0.62 | 0.54 to 0.71 | <0.001 |  | 0.66 | 0.58 to 0.76 | <0.001 |
|  |  | Nivolumab (BRAF+) | 0.72 | 0.53 to 0.97 | 0.033 |  | 0.7 | 0.51 to 0.95 | 0.021 |
|  | post-ipilimumab ≥2L | Nivolumab ≥2L | 0.61 | 0.41 to 0.90 | 0.014 |  | 0.53 | 0.36 to 0.79 | 0.002 |
|  |  | Pembrolizumab ≥2L | 0.44 | 0.28 to 0.69 | <0.001 |  | 0.39 | 0.26 to 0.60 | <0.001 |
| mUC | Cisplatin-ineligible | Atezolizumab 1L | 1.02 | 0.87 to 1.18 | .8 |  | 1.17 | 1.01 to 1.35 | 0.033 |
|  |  | Pembrolizumab | 0.83 | 0.70 to 0.97 | .021 |  | 0.84 | 0.73 to 0.97 | 0.018 |
|  | Post-platinum ≥2L | Atezolizumab ≥2L | 1.06 | 0.94 to 1.20 | .3 |  | 0.99 | 0.88 to 1.12 | 0.9 |
|  |  | Erdafitinib ≥2L | 1.2 | 0.82 to 1.76 | .4 |  | 1.11 | 0.74 to 1.65 | 0.6 |
|  |  | Nivolumab ≥2L | 0.79 | 0.65 to 0.96 | .016 |  | 0.85 | 0.71 to 1.03 | 0.1 |
|  | post-platinum and CIT ≥3L | Enfortumab vedotin-ejfv ≥3L | 0.89 | 0.68 to 1.15 | 0.4 |  | 0.75 | 0.59 to 0.96 | 0.025 |
| SCLC |  | Nivolumab ≥3L | 0.99 | 0.75 to 1.31 | >0.9 |  | 0.74 | 0.56 to 0.97 | 0.028 |

1L, first line; 2L, second line; 3L, third line; AA, accelerated approval; ALK, anaplastic lymphoma kinase; aNSCLC, advanced or metastatic non-small cell lung cancer; BRAF, v-raf murine sarcoma viral oncogene homolog B1; CI, confidence interval; CIT, cancer immunotherapy; EGFR, epidermal growth factor receptor; ER, estrogen receptor; fam-trastuzumab, fam-trastuzumab deruxtecan-nxki; HER2, human epidermal growth factor receptor-2; HR, hazard ratio; mBC, metastatic breast cancer; mUC, advanced or metastatic urethral cancer; OS, overall survival; rwPFS, real-world progression-free survival; SCLC, small cell lung cancer; SoC, standard of care.

**Supplementary Table S10.** Detailed analysis of outcomes for AA drugs in oncology solid tumors

| **Tumor** | **AA drug** | **PFS (months)** | | | | | **OS (months)** | |  | |  |
| --- | --- | --- | --- | --- | --- | --- | --- | --- | --- | --- | --- |
|  |  | **Weighted sample size, No.** | | **PFS outcomes** | | | **Weighted sample size, No.** | | **OS outcomes** | |  |
|  |  | **Cohort** | | **Cohort** | | **Difference** | **Cohort** | | **Cohort** | | **Difference** |
|  |  | **1** | **2** | **1** | **2** |  | **1** | **2** | **1** | **2** |  |
| aNSCLC | Alectinib ≥2L | 111 | 69 | 21.6 | 6.0 | 15.5 | 111 | 72 | 41.8 | 15.7 | 26.1 |
|  | Brigatinib ≥2L | 59 | 159 | 13.7 | 14.0 | –0.3 | 61 | 169 | 29.9 | 21.6 | 8.3 |
|  | Ceritinib ≥2L | 107 | 39 | 13.6 | 9.0 | 4.6 | 108 | 44.0 | 30.9 | 12.2 | 18.7 |
|  | Crizotinib | 123 | 600 | 16.6 | 9.3 | 7.3 | 127 | 619 | 36.1 | 18.1 | 18.0 |
|  | Lorlatinib ≥2L | 68 | 67 | 10.9 | 10.1 | 0.8 | 71 | 71 | 20.5 | 18.0 | 2.5 |
|  | Osimertinib | 161 | 570 | 13.9 | 8.1 | 5.7 | 171 | 598 | 29.1 | 19.2 | 9.9 |
|  | Pembrolizumab 1L | 666 | 5 081 | 15.5 | 11.3 | 4.2 | 699 | 5 241 | 23.1 | 22.7 | 0.4 |
|  | Pembrolizumab ≥2L | 40 | 60 | 12.7 | 9.4 | 3.3 | 42 | 62 | 25.2 | 24.3 | 0.9 |
| mBC | Atezolizumab (triple negative) | 133 | 1 229 | 11.5 | 8.2 | 3.4 | 134 | 1 252 | 22.6 | 17.8 | 4.8 |
|  | Fam-trastuzumab (HER2+) ≥3L | 171 | 397 | 15.8 | 10.8 | 5.0 | 175 | 405 | 26.0 | 22.4 | 3.6 |
|  | Palbociclib (ER+, HER2–) | 485 | 2 755 | 34.0 | 24.2 | 9.8 | 487 | 2 795 | 53.9 | 46.6 | 7.3 |
| Melanoma | Dabrafenib | 150 | 100 | 9.3 | 15.9 | –6.7 | 152 | 103 | 25.1 | 31.2 | –6.1 |
|  | Nivolumab plus ipilimumab | 396 | 1 428 | 35.0 | 19.9 | 15.1 | 407 | 1 462 | 43.1 | 31.4 | 11.7 |
|  | Nivolumab (BRAF+) | 64 | 613 | 28.9 | 16.5 | 12.4 | 64 | 625 | 45.0 | 30.3 | 14.7 |
|  | Nivolumab ≥2L | 65 | 61 | 28.9 | 10.2 | 18.7 | 67 | 63 | 45.5 | 19.6 | 25.9 |
|  | Pembrolizumab ≥2L | 81 | 35 | 27.1 | 3.0 | 24.1 | 84 | 36 | 40.5 | 9.6 | 30.9 |
| mUC | Atezolizumab 1L | 356 | 683 | 14.9 | 14.3 | 0.6 | 379 | 715 | 18.4 | 20.2 | –1.8 |
|  | Atezolizumab ≥2L | 467 | 928 | 11.2 | 13.6 | –2.4 | 485 | 955 | 20.2 | 19.9 | 0.3 |
|  | Enfortumab vedotin-ejfv ≥3L | 114 | 199 | 9.1 | 9.1 | –0.1 | 117 | 206 | 14.5 | 11.6 | 2.9 |
|  | Erdafitinib ≥2L | 55 | 74 | 6.2 | 12.4 | –6.2 | 55 | 75 | 14.1 | 19.3 | –5.2 |
|  | Nivolumab ≥2L | 988 | 152 | 19.2 | 13.3 | 5.9 | 157 | 1 022 | 22.0 | 19.7 | 2.3 |
|  | Pembrolizumab | 315 | 738 | 18.5 | 12.6 | 5.9 | 326 | 777 | 22.0 | 17.4 | 4.6 |
| SCLC | Nivolumab ≥3L | 74 | 284 | 6.7 | 3.2 | 3.5 | 73 | 292 | 11.4 | 5.9 | 5.5 |

1L, first line; 2L, second line; 3L, third line; AA, accelerated approval; aNSCLC, advanced non-small cell lung cancer; BRAF, v-raf murine sarcoma viral oncogene homolog B1; ER, estrogen receptor; fam-trastuzumab, fam-trastuzumab deruxtecan-nxki; HER2, human epidermal growth factor receptor-2; mBC, metastatic breast cancer; mUC, advanced or metastatic urethral cancer; OS, overall survival; PFS, progression-free survival; SCLC, small cell lung cancer.
