# Supplementary material for: Real-World Clinical Oncology Outcomes Associated with the Accelerated Approval Pathway
Source: Cancer Res Commun. 2026 Jan 23;6(1):191–200. doi: 10.1158/2767-9764.CRC-25-0225 (PMC12828896; doi:10.1158/2767-9764.CRC-25-0225)
Supplement: Supplementary Table S9 — Table S9. Unweighted risk of progression or death and risk of death for AA drugs vs SoC [file crc-25-0225_supplementary_table_s9_suppst9.docx]

## **Supplementary Table S9.** Unweighted risk of progression or death and risk of death for AA drugs vs SoC

|  |  |  |  | **rwPFS** |  |  |  | **OS** |  |
| --- | --- | --- | --- | --- | --- | --- | --- | --- | --- |
|  |  |  | **HR** | **95% CI** | ***P*** |  | **HR** | **95% CI** | ***P*** |
| aNSCLC | ALK+ | Alectinib ≥2L | 0.32 | 0.23 to 0.46 | <0.001 |  | 0.28 | 0.20 to 0.40 | <0.001 |
|  |  | Brigatinib ≥2L | 0.89 | 0.63 to 1.26 | 0.5 |  | 0.68 | 0.48 to 0.97 | 0.035 |
|  |  | Ceritinib ≥2L | 0.41 | 0.28 to 0.62 | <0.001 |  | 0.44 | 0.30 to 0.63 | <0.001 |
|  |  | Crizotinib | 0.6 | 0.48 to 0.75 | <0.001 |  | 0.4 | 0.32 to 0.50 | <0.001 |
|  |  | Lorlatinib ≥2L | 0.83 | 0.56 to 1.21 | 0.3 |  | 0.77 | 0.52 to 1.14 | 0.2 |
|  | CIT | Pembrolizumab 1L | 0.8 | 0.73 to 0.88 | <0.001 |  | 0.99 | 0.91 to 1.09 | >0.9 |
|  |  | Pembrolizumab ≥2L | 0.89 | 0.58 to 1.35 | 0.6 |  | 1 | 0.66 to 1.53 | >0.9 |
|  | EGFR+ | Osimertinib | 0.66 | 0.55 to 0.78 | <0.001 |  | 0.66 | 0.56 to 0.78 | <0.001 |
| mBC | 1L | Atezolizumab (triple negative) | 0.8 | 0.66 to 0.98 | 0.029 |  | 0.73 | 0.59 to 0.91 | 0.004 |
|  |  | Palbociclib (ER+, HER2–) | 0.73 | 0.65 to 0.82 | <0.001 |  | 0.77 | 0.69 to 0.87 | <0.001 |
|  | ≥3L | Fam-trastuzumab (HER2+) ≥3L | 0.61 | 0.49 to 0.76 | <0.001 |  | 0.77 | 0.61 to 0.97 | 0.027 |
| Melanoma | BRAF+ | Dabrafenib | 0.97 | 0.73 to 1.28 | 0.8 |  | 0.96 | 0.72 to 1.27 | 0.8 |
|  | 1L | Nivolumab plus ipilimumab | 0.62 | 0.54 to 0.71 | <0.001 |  | 0.66 | 0.58 to 0.76 | <0.001 |
|  |  | Nivolumab (BRAF+) | 0.72 | 0.53 to 0.97 | 0.033 |  | 0.7 | 0.51 to 0.95 | 0.021 |
|  | post-ipilimumab ≥2L | Nivolumab ≥2L | 0.61 | 0.41 to 0.90 | 0.014 |  | 0.53 | 0.36 to 0.79 | 0.002 |
|  |  | Pembrolizumab ≥2L | 0.44 | 0.28 to 0.69 | <0.001 |  | 0.39 | 0.26 to 0.60 | <0.001 |
| mUC | Cisplatin-ineligible | Atezolizumab 1L | 1.02 | 0.87 to 1.18 | .8 |  | 1.17 | 1.01 to 1.35 | 0.033 |
|  |  | Pembrolizumab | 0.83 | 0.70 to 0.97 | .021 |  | 0.84 | 0.73 to 0.97 | 0.018 |
|  | Post-platinum ≥2L | Atezolizumab ≥2L | 1.06 | 0.94 to 1.20 | .3 |  | 0.99 | 0.88 to 1.12 | 0.9 |
|  |  | Erdafitinib ≥2L | 1.2 | 0.82 to 1.76 | .4 |  | 1.11 | 0.74 to 1.65 | 0.6 |
|  |  | Nivolumab ≥2L | 0.79 | 0.65 to 0.96 | .016 |  | 0.85 | 0.71 to 1.03 | 0.1 |
|  | post-platinum and CIT ≥3L | Enfortumab vedotin-ejfv ≥3L | 0.89 | 0.68 to 1.15 | 0.4 |  | 0.75 | 0.59 to 0.96 | 0.025 |
| SCLC |  | Nivolumab ≥3L | 0.99 | 0.75 to 1.31 | >0.9 |  | 0.74 | 0.56 to 0.97 | 0.028 |

1L, first line; 2L, second line; 3L, third line; AA, accelerated approval; ALK, anaplastic lymphoma kinase; aNSCLC, advanced or metastatic non-small cell lung cancer; BRAF, v-raf murine sarcoma viral oncogene homolog B1; CI, confidence interval; CIT, cancer immunotherapy; EGFR, epidermal growth factor receptor; ER, estrogen receptor; fam-trastuzumab, fam-trastuzumab deruxtecan-nxki; HER2, human epidermal growth factor receptor-2; HR, hazard ratio; mBC, metastatic breast cancer; mUC, advanced or metastatic urethral cancer; OS, overall survival; rwPFS, real-world progression-free survival; SCLC, small cell lung cancer; SoC, standard of care.
