# Supplementary material for: Real-World Clinical Oncology Outcomes Associated with the Accelerated Approval Pathway
Source: Cancer Res Commun. 2026 Jan 23;6(1):191–200. doi: 10.1158/2767-9764.CRC-25-0225 (PMC12828896; doi:10.1158/2767-9764.CRC-25-0225)
Supplement: Supplementary Table S10 — Table S10. Detailed analysis of outcomes for AA drugs in oncology solid tumors [file crc-25-0225_supplementary_table_s10_suppst10.docx]

**Supplementary Table S10.** Detailed analysis of outcomes for AA drugs in oncology solid tumors

| **Tumor** | **AA drug** | **PFS (months)** | | | | | **OS (months)** | |  | |  |
| --- | --- | --- | --- | --- | --- | --- | --- | --- | --- | --- | --- |
|  |  | **Weighted sample size, No.** | | **PFS outcomes** | | | **Weighted sample size, No.** | | **OS outcomes** | |  |
|  |  | **Cohort** | | **Cohort** | | **Difference** | **Cohort** | | **Cohort** | | **Difference** |
|  |  | **1** | **2** | **1** | **2** |  | **1** | **2** | **1** | **2** |  |
| aNSCLC | Alectinib ≥2L | 111 | 69 | 21.6 | 6.0 | 15.5 | 111 | 72 | 41.8 | 15.7 | 26.1 |
|  | Brigatinib ≥2L | 59 | 159 | 13.7 | 14.0 | –0.3 | 61 | 169 | 29.9 | 21.6 | 8.3 |
|  | Ceritinib ≥2L | 107 | 39 | 13.6 | 9.0 | 4.6 | 108 | 44.0 | 30.9 | 12.2 | 18.7 |
|  | Crizotinib | 123 | 600 | 16.6 | 9.3 | 7.3 | 127 | 619 | 36.1 | 18.1 | 18.0 |
|  | Lorlatinib ≥2L | 68 | 67 | 10.9 | 10.1 | 0.8 | 71 | 71 | 20.5 | 18.0 | 2.5 |
|  | Osimertinib | 161 | 570 | 13.9 | 8.1 | 5.7 | 171 | 598 | 29.1 | 19.2 | 9.9 |
|  | Pembrolizumab 1L | 666 | 5 081 | 15.5 | 11.3 | 4.2 | 699 | 5 241 | 23.1 | 22.7 | 0.4 |
|  | Pembrolizumab ≥2L | 40 | 60 | 12.7 | 9.4 | 3.3 | 42 | 62 | 25.2 | 24.3 | 0.9 |
| mBC | Atezolizumab (triple negative) | 133 | 1 229 | 11.5 | 8.2 | 3.4 | 134 | 1 252 | 22.6 | 17.8 | 4.8 |
|  | Fam-trastuzumab (HER2+) ≥3L | 171 | 397 | 15.8 | 10.8 | 5.0 | 175 | 405 | 26.0 | 22.4 | 3.6 |
|  | Palbociclib (ER+, HER2–) | 485 | 2 755 | 34.0 | 24.2 | 9.8 | 487 | 2 795 | 53.9 | 46.6 | 7.3 |
| Melanoma | Dabrafenib | 150 | 100 | 9.3 | 15.9 | –6.7 | 152 | 103 | 25.1 | 31.2 | –6.1 |
|  | Nivolumab plus ipilimumab | 396 | 1 428 | 35.0 | 19.9 | 15.1 | 407 | 1 462 | 43.1 | 31.4 | 11.7 |
|  | Nivolumab (BRAF+) | 64 | 613 | 28.9 | 16.5 | 12.4 | 64 | 625 | 45.0 | 30.3 | 14.7 |
|  | Nivolumab ≥2L | 65 | 61 | 28.9 | 10.2 | 18.7 | 67 | 63 | 45.5 | 19.6 | 25.9 |
|  | Pembrolizumab ≥2L | 81 | 35 | 27.1 | 3.0 | 24.1 | 84 | 36 | 40.5 | 9.6 | 30.9 |
| mUC | Atezolizumab 1L | 356 | 683 | 14.9 | 14.3 | 0.6 | 379 | 715 | 18.4 | 20.2 | –1.8 |
|  | Atezolizumab ≥2L | 467 | 928 | 11.2 | 13.6 | –2.4 | 485 | 955 | 20.2 | 19.9 | 0.3 |
|  | Enfortumab vedotin-ejfv ≥3L | 114 | 199 | 9.1 | 9.1 | –0.1 | 117 | 206 | 14.5 | 11.6 | 2.9 |
|  | Erdafitinib ≥2L | 55 | 74 | 6.2 | 12.4 | –6.2 | 55 | 75 | 14.1 | 19.3 | –5.2 |
|  | Nivolumab ≥2L | 988 | 152 | 19.2 | 13.3 | 5.9 | 157 | 1 022 | 22.0 | 19.7 | 2.3 |
|  | Pembrolizumab | 315 | 738 | 18.5 | 12.6 | 5.9 | 326 | 777 | 22.0 | 17.4 | 4.6 |
| SCLC | Nivolumab ≥3L | 74 | 284 | 6.7 | 3.2 | 3.5 | 73 | 292 | 11.4 | 5.9 | 5.5 |

1L, first line; 2L, second line; 3L, third line; AA, accelerated approval; aNSCLC, advanced non-small cell lung cancer; BRAF, v-raf murine sarcoma viral oncogene homolog B1; ER, estrogen receptor; fam-trastuzumab, fam-trastuzumab deruxtecan-nxki; HER2, human epidermal growth factor receptor-2; mBC, metastatic breast cancer; mUC, advanced or metastatic urethral cancer; OS, overall survival; PFS, progression-free survival; SCLC, small cell lung cancer.
